# Human Fibroblast‐Derived Matrix Hydrogel Accelerates Regenerative Wound Remodeling Through the Interactions with Macrophages

**DOI:** 10.1002/advs.202305852

**Published:** 2024-03-12

**Authors:** Cininta Savitri, Sang Su Ha, Jae Won Kwon, Sung Hoon Kim, Young‐Min Kim, Hyun Mee Park, Haejin Kwon, Mi Jung Ji, Kwideok Park

**Affiliations:** ^1^ Center for Biomaterials Korea Institute of Science and Technology (KIST) Seoul 02792 Republic of Korea; ^2^ Division of Bio‐Medical Science and Technology, KIST School University of Science and Technology (UST) Seoul 02792 Republic of Korea; ^3^ Advanced Analysis and Data Center KIST Seoul 02792 Republic of Korea

**Keywords:** cell‐derived, decellularized extracellular matrix, human fibroblast‐derived matrix hydrogel, macrophages, matrix‐macrophages interaction, wound healing

## Abstract

Herein, a novel extracellular matrix (ECM) hydrogel is proposed fabricated solely from decellularized, human fibroblast‐derived matrix (FDM) toward advanced wound healing. This FDM‐gel is physically very stable and viscoelastic, while preserving the natural ECM diversity and various bioactive factors. Subcutaneously transplanted FDM‐gel provided a permissive environment for innate immune cells infiltration. Compared to collagen hydrogel, excellent wound healing indications of FDM‐gel treated in the full‐thickness wounds are noticed, particularly hair follicle formation via highly upregulated β‐catenin. Sequential analysis of the regenerated wound tissues disclosed that FDM‐gel significantly alleviated pro‐inflammatory cytokine and promoted M2‐like macrophages, along with significantly elevated vascular endothelial growth factor (VEGF) and basic fibroblast growth factor (bFGF) level. A mechanistic study demonstrated that macrophages‐FDM interactions through cell surface integrins α5β1 and α1β1 resulted in significant production of VEGF and bFGF, increased Akt phosphorylation, and upregulated matrix metalloproteinase‐9 activity. Interestingly, blocking such interactions using specific inhibitors (ATN161 for α5β1 and obtustatin for α1β1) negatively affected those pro‐healing growth factors secretion. Macrophages depletion animal model significantly attenuated the healing effect of FDM‐gel. This study demonstrates that the FDM‐gel is an excellent immunomodulatory material that is permissive for host cells infiltration, resorbable with time, and interactive with macrophages, where it thus enables regenerative matrix remodeling toward a complete wound healing.

## Graphical abstract

A novel extracellular matrix hydrogel (FDM‐gel) is fabricated solely from decellularized, human fibroblast‐derived matrix. FDM‐gel is physically stable and preserves the natural ECM diversity with various bioactive factors. As an immunomodulatory material, FDM‐gel provides a permissive environment for cells infiltration, allows significantly lower inflammation, and promotes M2‐like macrophages, which thus enables regenerative matrix remodeling toward a complete wound healing.

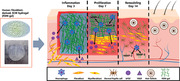

## 1 Introduction

The skin is a forefront defense line that protects us from pathogens and numerous external stimuli. Skin injury leads to loss of water and electrolytes, secondary infection, and multiple complications.^[^ ^1^, ^2^ ^]^ Simple acute wounds heal naturally with time. Deep and chronic wounds, however, are still challenging to be fully regenerated without scar formation or recurrence, because they do not follow the natural wound healing process. In fact, wound repair is a very complex process that involves many diverse participants, including immune cells, growth factors, cytokines, neighboring cells, and the extracellular matrix (ECM).^[^ ^3^, ^4^ ^]^ To this end, deep understanding of such coordinated and dynamic interplays has been a critical issue for complete wound healing.

Traditionally, the role of immune cells in tissue regeneration was largely unknown and often underestimated. Fortunately, the rapidly growing interest in immune cells makes them a pivotal element in successful tissue regeneration.^[5]^ For example, macrophages, an innate immune cell, are extremely plastic in their phenotype and play a crucial role in initiating, modulating, and completing the whole process of wound healing.^[^ ^6^, ^7^ ^]^ Accordingly, many basic sciences pursue better understanding of the mechanism of macrophage functions. In parallel, engineers would learn the working principle of macrophages and attempt to modulate the macrophage phenotype. The common strategy is to induce macrophage polarization shift from pro‐inflammatory M1‐like to anti‐inflammatory M2‐like phenotype, where material‐based approaches include surface chemistry, surface topography, new materials, and nanoparticles (NPs).^[^ ^8^, ^9^, ^10^ ^]^ Furthermore, molecular strategies target specific immune responses using protease inhibitors, cytokines, miRNA, small interfering RNA and extracellular vesicles.^[^ ^11^, ^12^, ^13^ ^]^ Meanwhile, ECM also has a very important role in tissue regeneration, where ECM would interact with cells and thereby regulate cellular functions, including macrophages.^[^ ^14^, ^15^, ^16^ ^]^ Decellularized ECM obtained from the animal organs or tissues has thus been widely harnessed as an attractive resource in regenerative medicine, due mainly to its native tissue mimicking ECM characteristics.^[17]^ Many documents have found dECM promising in treating skin wounds.^[^ ^10^, ^18^, ^19^ ^]^

In this study, we propose a novel hydrogel, fibroblast‐derived matrix hydrogel (FDM‐gel) that is directly fabricated using cell‐derived, decellularized ECM (cdECM) obtained from in vitro‐cultured human fibroblasts. This FDM‐gel is physically stable, viscoelastic, and thus shows a hydrogel‐like behavior with an extremely high water content. To the best of our knowledge, this FDM‐gel is the first ECM hydrogel made of 100% human fibroblast‐secreted ECM components without the addition of crosslinking agents, synthetic or natural polymers. The pro‐healing cytokines and growth factors embedded in the FDM‐gel was another notable benefit for tissue regeneration. Our FDM‐gel was also fully resorbable over time, interactive with the host cells, and showed immunomodulatory properties. In particular, the role of macrophages in wound healing was of primary interest. Host macrophages actively interacted with the transplanted FDM‐gel and such interactions enable the regenerative remodeling of the FDM‐gel. Here, we thoroughly assessed the therapeutic efficacy of FDM‐gel and its underlying mechanism, particularly targeting the interactions between macrophage and FDM.

## 2 Results and Discussion

### 2.1 Fabrication of FDM‐Gel and Characterizations

**Figure 1:**
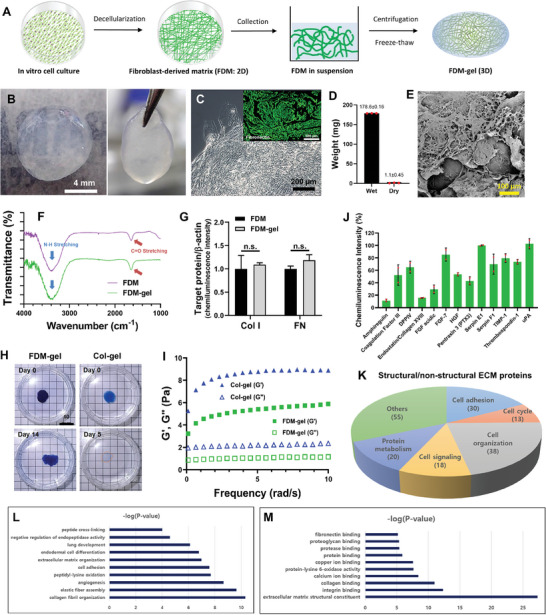
Fabrication and characterization of FDM‐gel. A) Fabrication process of FDM‐gel. B) Appearance of the FDM‐gel and the one gripped by forceps. C) Microscopic view of FDM‐gel (Inset: immunofluorescence of fibronectin). D) Wet and dry weight of FDM‐gel. E) Surface texture of FDM‐gel as observed via SEM. F) Comparison of the functional groups between FDM (2D) and FDM‐gel (3D) as assessed via FT‐IR. G) Major proteins (collagen and fibronectin) in the FDM and FDM‐gel, respectively via western blot. H) Examination of physical stability of FDM‐gel and Col‐gel as stained by coomassie blue and then subsequently incubated at 37 °C for up to 14 days. I) Rheological property of FDM gel and Col‐gel. J) Identification of bioactive factors contained in the FDM‐gel via human angiogenic factors array. K) Proteomic analysis of FDM‐gel: structural and non‐structural proteins as assessed via mass spectrometer coupled with nano‐LC system. L) Gene ontology (GO) functional analysis: statistically significant top 10 biological processes. M) GO functional analysis: statistically significant top 10 molecular functions.

For the preparation of FDM‐gel, human fibroblasts were cultivated on the culture plastic, then subject to a mild decellularization, followed by collection of the cdECM, centrifugation, and finally freeze‐thawing (**Figure** **1A**). These processes packed the ECM components and physically united numerous ECM molecules together, causing proteins‐proteins interaction and eventually generating a pure ECM hydrogel, i.e., FDM‐gel (Figure 1B). The protein content of single FDM‐gel was estimated to 760±54.5 µg mL^−1^ via bicinchoninic acid (BCA) assay. Our FDM‐gel retained a good mechanical stability that enabled shape maintenance when gripped by using forceps. Phase contrast image showed an interconnected fibrous matrix in the FDM‐gel (Figure 1C) and immunofluorescence disclosed a major ECM component, fibronectin (green) (Figure 1C, Inset). Hydrophilic FDM‐gel reserved extremely high water content, as determined by the difference between wet and dried weight (Figure 1D). The scanning electron microcope (SEM) image exhibited a fibrous mesh network on the surface of FDM‐gel (Figure 1E). Since the FDM‐gel (3D) was fabricated from 2D FDM, FT‐IR confirmed the same functional groups between FDM and FDM‐gel (Figure 1F), as assessed by the typical peaks of proteins, such as C = O bond (1500–1700 cm^−1^) and N‐H bond (3300–3500 cm^−1^). Western blot analysis of major ECM proteins (collagen I, fibronectin) also suggested little compositional difference between them (Figure 1G). Interestingly, opposed to the general notion of ECM, FDM‐gel showed an excellent physical stability. FDM‐gel pre‐stained by coomassie blue maintained its original shape at 37 °C in PBS solution, where it could keep blue stains for up to 14 day, along with a barely swelling property (Figure 1H). This result was sharply contrasted with collagen hydrogel (Col‐gel) that lost the blue color on day 5. As compared to Col‐gel, bare leakage of coomassie blue stains indicated a tight network of ECM molecules and/or ECM proteins‐dye binding. FDM‐gel was viscoelastic. The mechanical property of FDM‐gel, as assessed by storage (G′) and loss modulus (G″) was half that of Col‐gel (Figure 1I). Both Col‐gel and FDM‐gel disclosed significantly different internal structure, where FDM had larger pore size (70±5 µm) but Col‐gel showed rather more compact morphology, with the pore size of 25±5 µm (Figure S1A, Supporting Information). Individual ECM components that interacted with each other may have enabled our FDM‐gel to build up a resilient property during freeze‐thawing process, in which no chemicals were employed. It is notable that more cell infiltration inside FDM‐gel is due mainly to the difference in pore size rather than the immunomodulatory properties of FDM. Reservation of bioactive molecules was also another huge benefit of FDM‐gel regarding tissue regeneration. Angiogenic‐related cytokines array disclosed diverse bioactive molecules contained in the FDM‐gel, such as bFGF, FGF‐7, hepatocyte growth factor (HGF), and others (Figure 1J; Figure S1B, Supporting Information). Originated from the human fibroblasts, those biomolecules could survive from current decellularization process and remain embedded in the FDM‐gel, due mainly to the mild treatment protocol we applied. Moreover, proteomic analysis of FDM‐gel uncovered many structural and non‐structural ECM proteins that were involved in various cellular functions, such as cell cycle, adhesion, signaling, and organization (Figure 1K). This supported physiologically relevant diversity of ECM constituents in our FDM‐gel. Gene ontology (GO) functional analysis elucidated significant enrichments in biological processes as well as in molecular functions. For wound healing associated biological processes, collagen fibril organization, angiogenesis and cell adhesions are among statistically significant top 10 biological processes (Figure 1L). And ECM structural constituents, integrin bindings and collagen bindings are among statistically significant top 10 molecular functions (Figure 1M). Taken together, our FDM‐gel is a complex of biophysical entity (ECM) and bioactive molecules, where each has specific role but they are equally important in wound healing.

### 2.2 FDM‐Gel Allowed Active Infiltration of the Host Cells in the Subcutaneous Environment

**Figure 2:**
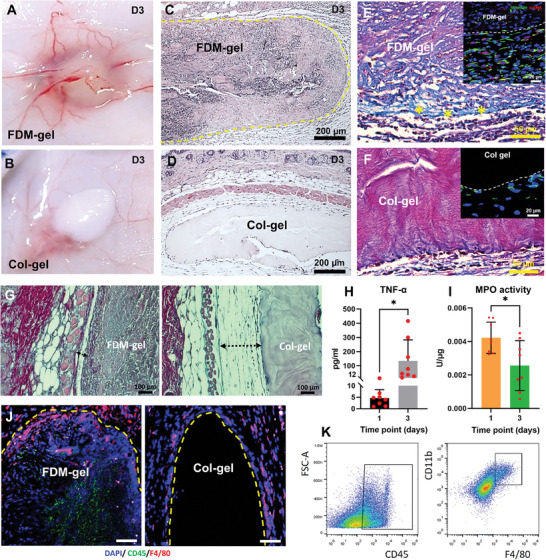
Subcutaneous transplantation of FDM‐gel and Col‐gel, respectively. Appearance of A) FDM‐gel and B) Col‐gel at 3 day post‐transplantation subcutaneously. C) Cross‐sectional view of C) FDM‐gel and D) Col‐gel at 3 day via H&E staining. Histological analysis of E) FDM‐gel and F) Col‐gel as assessed via Herovici staining (inset: co‐immunofluorescence via vimentin (green) and α‐SMA (red), along with DAPI staining) (*: the interface between FDM‐gel and host tissue). The dotted line (Insets) is the interface between host tissue and FDM‐gel or Col‐gel. G) Fibrous layer formation in the subcutaneous FDM‐gel and Col‐gel, respectively. Measurement of H) tumor necrosis factor (TNF)‐α and I) MPO activity in the FDM‐gel harvested at 1 and 3 day, respectively. J) Identification of the macrophages distributed inside/ around FDM‐gel and Col‐gel at 3 day, as assessed via immunofluorescence of CD45 and F4/80, along with DAPI staining. K) FACS analysis of the innate immune cells infiltrated inside the FDM‐gel, as determined by the antibody of leucocytes (CD45^+^) and macrophages (CD11b^+^/F4/80^+^). Statistically significant difference: *p<0.05.

For successful tissue regeneration, transplanted scaffolds must be replaced with new tissues in a timely manner. To this end, tissue engineered scaffold should be designed to allow cells to actively remodel the scaffold on their own. One strategy to achieve this goal is host cells recruitment, especially innate immune cells inside the scaffold, because their role is crucial in tissue regeneration but still poorly understood. More importantly, such immune cells infiltration must be followed by a series of coordinated operations toward wound tissue regeneration. In the next experiment, we assessed the in vivo response to FDM‐gel, where we inserted FDM‐gel and Col‐gel (a control) subcutaneously in the mice, respectively. A gross observation found a notable difference between the two gels at 3 day: highly vascularized appearance around FDM‐gel but no such phenomenon with Col‐gel (**Figure** **2**A,B). Interestingly, we also learned a sharp contrast, in terms of the recruited cells population. From H&E staining, we noticed many cells inside the FDM‐gel, suggesting active cellular infiltration from the host cells (Figure 2C). However, there were few cells in the Col‐gel (Figure 2D). We speculate that active cellular infiltration with FDM‐gel is due mainly to the combined effects, which are larger pore size (Figure S1A, Supporting Information) and chemoattractive factors embedded in FDM‐gel (Figure 1J). Herovici staining showed large percentage of young collagen fibers (light blue) in the FDM‐gel and also at the boundary (yellow asterisks) (Figure 2E), along with large number of vimentin (+) and α‐SMA (+) cells in the host tissue domain (Figure 2E, Inset). The Col‐gel was stained in purple red, which is an indicative of mature collagen (Figure 2F) and showed small number of cells at the interface (Figure 2F, Inset). In addition, evaluation of a fibrous barrier around the transplants showed a relatively thin fibrous layer with FDM‐gel than that of Col‐gel (Figure 2G). The exact reason of this phenomenon is not simple. One of them we postulate is a unique property, i.e., immunomodulatory one that FDM‐gel provides. We also quantitatively assessed inflammation‐related factors, specifically TNF‐α and myeloperoxidase (MPO), both of which are secreted from innate immune cells, such as macrophages and neutrophils. The concentration of TNF‐α in the FDM‐gel was negligible at 1 day and increased (but still very low level) at 3 day (Figure 2H). MPO activity significantly decreased with time (Figure 2I). Moreover, as a direct evidence of infiltrated immune cells, we noticed cluster of differentiation (CD)11b^+^/F4/80^+^ cells inside the FDM‐gel via immunofluorescence, while Col‐gel showed few positive cells (Figure 2J). FACS analysis also revealed the immune cells (CD45^+^), accounting for 44.3±11.7% of the total cells population. Macrophages (CD11b^+^/F4/80^+^) consisted of 8.64±2.38% out of those immune cells (Figure 2K). Our following investigations strongly indicated that such cellular infiltration into the FDM‐gel should be interpreted as an early, positive sign of tissue regeneration, while the mechanism should be of particular interest in the future study.

### 2.3 FDM‐Gel Promoted a Complete Full‐Thickness Skin Wound Healing

**Figure 3:**
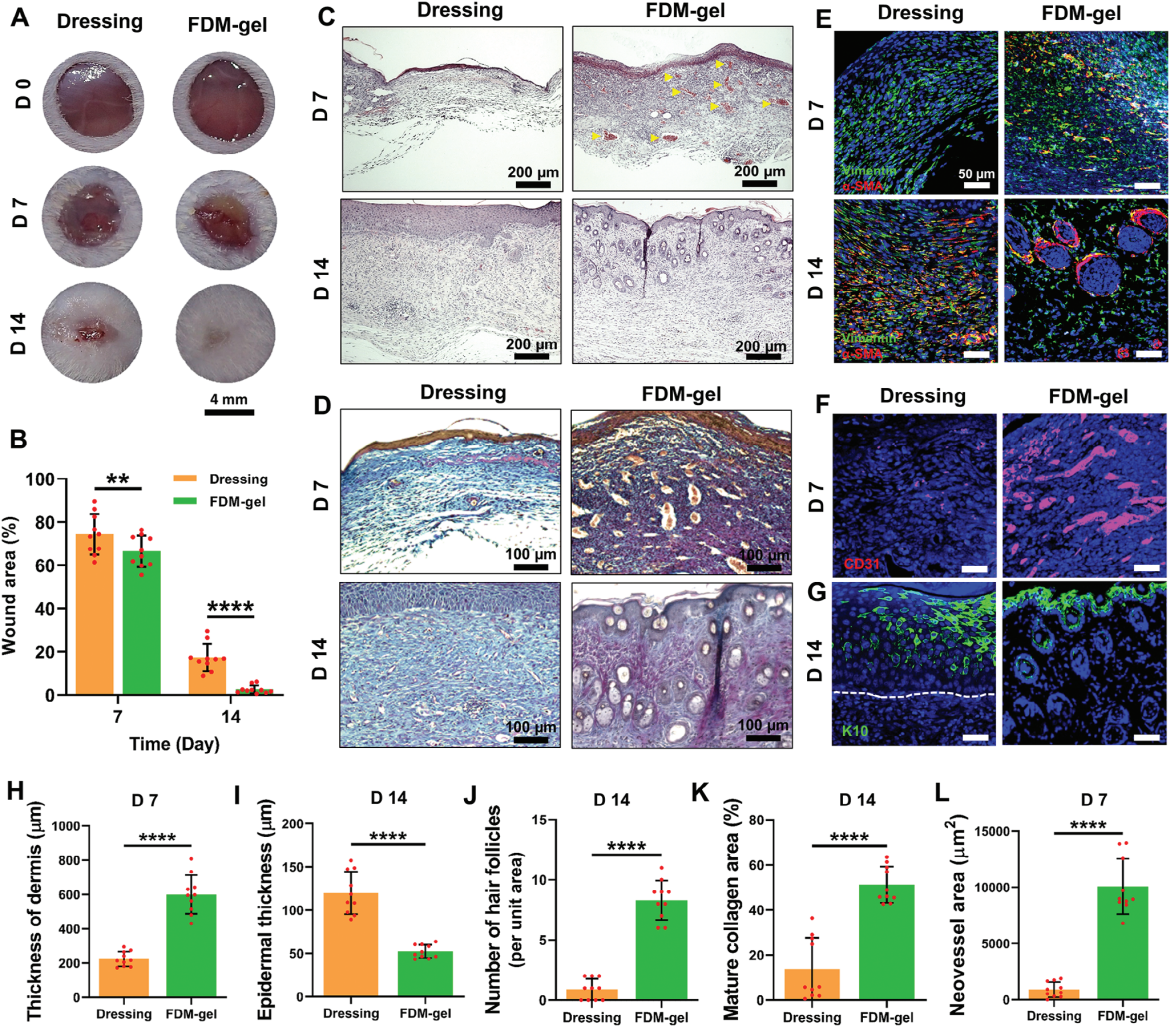
Transplantation of FDM‐gels into excisional, full‐thickness skin wounds and comprehensive assessment of wound healing efficacy. A) Gross appearance of the wounds treated with Tegaderm dressing or FDM‐gel at 7 and 14 day. B) Quantitative measurement of the wound areas with time. C) Histological analysis of the wound regions (epidermis and dermis) via H&E staining (Yellow triangles in the FDM‐gel indicate new blood vessels). D) Collagen deposition and the degree of maturation in the regenerating wounds as assessed via Herovici staining. Immature and mature collagen appears in blue and purple/red, respectively. E) Co‐immunofluorescence staining of the wound area via vimentin and α‐SMA, along with DAPI staining. F) CD31 immunostaining for the endothelial cells at 7 day. G) Assessment of epidermal keratinocyte via K10 staining at 14 day. Quantitative analysis of wound healing parameters. H) Thickness of dermis (µm) at 7 day. I) The epidermal thickness (µm) at 14 day. J) Number of hair follicles per unit area at 14 day. K) Mature collagen area (%) at 14 day. L) Neovessel area (µm^2^) at 7 day. Statistically significant difference: **p<0.01 or ****p<0.0001.

We investigated therapeutic efficacy of FDM‐gel via full‐thickness, excisional skin wound model. When either FDM‐gel or Tegaderm dressing (a control) was administered in the wounds, gross appearance of wound sites exhibited much faster wound closure with FDM‐gel, which was just single treatment during the entire experimental period (**Figure** **3**A). This was supported by quantitatively measured wound area (%) at 7 and 14 day, in which the difference was statistically significant between the two treatments (Figure 3B). For more detailed analysis, when the wound tissues were examined, both dressing and FDM‐gel group showed underdeveloped epidermis at 7 day (Figure 3C, Top and Figure S4A, Supporting Information). However, the regenerated dermis was greatly thicker in the FDM‐gel treated wounds on day 7 than that of dressing (Figure 3C, Top and Figure 3H). We also noticed that the epidermis thickness was significantly thinner (Figure 3I) and found many hair follicles in the dermis at 14 day (Figure 3C, Bottom) when treated with FDM‐gel. This result was confirmed by quantitative assessment of new hair follicles per unit area of dermis (Figure 3J). Moreover, we prepared a full range of the original wound image as stained via hematoxylin and eosin (H&E) and immunofluorescence, respectively (Figure S4B,C, Supporting Information). We then noticed the panniculus carnosus muscles that were still impaired and not fully regenerated at 14 day. Therefore, our results suggested new hair follicles formation in the apparent wound bed. Herovici staining tells the difference between immature (blue) and mature collagen (purple red). Deposition of mature collagen was obvious at 14 day with FDM‐gel treatment, while there was still immature collagen matrix in the dressing group (Figure 3D). We quantitatively analyzed the area of mature collagen deposition and learned a significant difference between them (Figure 3K). Build‐up of mature collagen with time is apparently a good sign of wound repair, instead of immature young collagen. Meanwhile, we paid much attention to the role of myofibroblasts during wound healing process. Co‐immunostaining of vimentin and α‐SMA disclosed that myofibroblastic cells were abundant at the early stage (7 day) in the FDM‐gel but few cells were found in the dressing group (Figure 3E). Interestingly, this trend was reversed at 14 day, where the myofibroblastic cells were belatedly populous in the dressing‐treated wounds but few ones were detected in the FDM‐gel group. Our results suggested that the FDM‐gel treated wounds followed a right path of wound repair in a time‐dependent manner. Since wound angiogenesis is another essential part of tissue repair, we also observed significantly higher level of neovascularization in the regenerated dermis when treated with FDM‐gel at 7 day as assessed via H&E (marked in yellow triangles) (Figure 3C), CD31 immunostaining (Figure 3F), and neovessels formation per unit area (Figure 3L). FDM‐gel had pro‐angiogenic activity, where the endothelial cells contained in the FDM‐gel were homogeneously distributed (Figure S2A,B, Supporting Information) and displayed a tube‐like structure formation at 7 day (Figure S2C–E, Supporting Information). Additionally, active recruitment of blood vessels around the FDM‐gel in the subcutaneous milieu (Figure 2A) further demonstrated the pro‐angiogenic activity. Examination of epidermis layer using keratin 10 (K10) presented a well‐developed epidermal layer with the FDM‐gel treatment (Figure 3G). In a comparison study, we also evaluated the wound healing efficacy between FDM‐gel and Col‐gel using the same wound model (Figure S3A, Supporting Information). The difference of wound area (%) was notable at 14 day (Figure S3B, Supporting Information). Our FDM‐gel possessed significantly advanced capability in the wound repair over Col‐gel, as assessed via histology (H&E, Herovici staining) (Figure S3C,D, Supporting Information) and quantitative analysis of each wound healing parameter (Figure S3H–L, Supporting Information). In addition, Col‐gel treated wounds revealed significantly higher population of myofibroblast at later time point (D14) than that of FDM‐gel (Figure S3E, Supporting Information). Moreover, Col‐gel treatment presented significantly weak CD31 signals in the dermis (Figure S3F, Supporting Information) and also exhibited an immature epidermis layer at 14 day as assessed via K10 immunostaining (Figure S3G, Supporting Information). These results clearly showed that pro‐regenerative property of FDM‐gel was superior to Col‐gel. To this end, we excluded Col‐gel in the following investigations and concentrated more on the wound healing capability of FDM‐gel, along with the control group (Tegaderm dressing).

### 2.4 FDM‐Gel Stimulated Hair Follicles Formation Through Upregulated β‐Catenin and Epithelial Cells Migration

**Figure 4:**
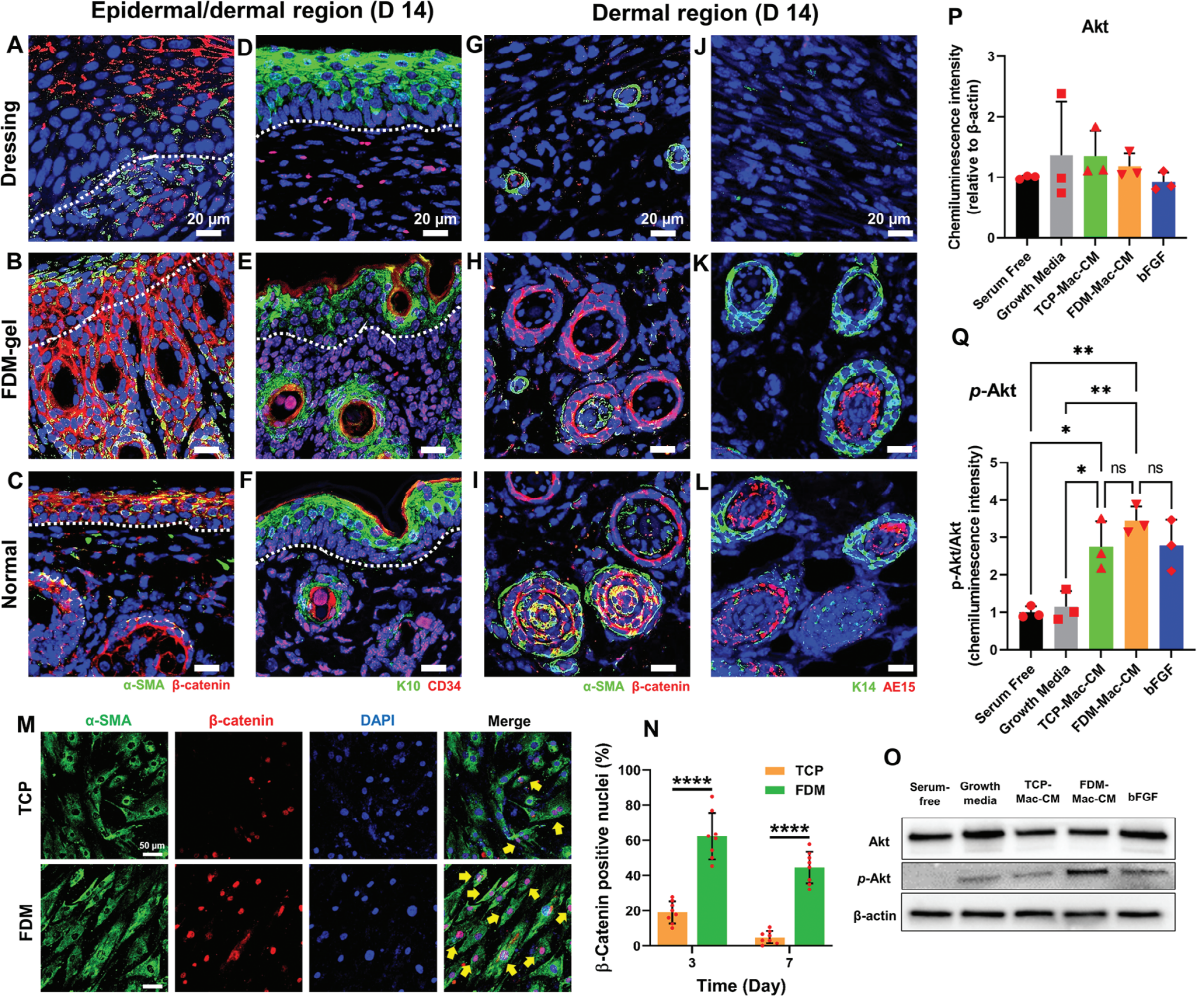
Hair follicles formation and interaction between FDM and human dermal papilla cells in vitro. Co‐immunofluorescence (β‐catenin and α‐SMA) staining in the epidermal region at 14 day, where the wounds were treated with A) dressing or B) FDM‐gel. Co‐immunofluorescence (K10 and CD34) of the epidermal region, when treated with D) dressing or E) FDM‐gel. C,F) The normal skin tissue serves as a positive control. Co‐immunofluorescence (β‐catenin and α‐SMA) in the dermal region at 14 day, in which the wounds were administered with G) dressing or H) FDM‐gel. Co‐immunofluorescence (K14 and AE15) in the dermal region, when treated with J) dressing or K) FDM‐gel. I,L) The normal skin tissue was co‐stained as well. M) HDPCs were cultivated on either TCP or FDM and examined for the expression of α‐SMA and β‐catenin at 3 day. N) Quantitative measurement of β‐catenin (+) cells and their comparison between TCP and FDM substrate. O) HDPCs were treated with macrophage‐derived conditioned media (TCP‐Mac‐CM and FDM‐Mac‐CM), where macrophages were grown on TCP or FDM and assessed via western blot for Akt and phosphorylated Akt (*p*‐Akt). Serum‐free and growth media are negative control, whereas b‐FGF (700 pg mL^−1^) is a positive control. Quantitative comparison of P) Akt and Q) *p*‐Akt level. Statistically significant difference: *p<0.05, **p<0.01 or ****p<0.0001.

Full recovery of skin appendages (i.e., hair follicle, glands) is a crucial step in the wound regeneration. We spotted many hair follicles that were newly formed in the dermis when treated with FDM‐gel, as assessed via histological staining (Figure 3C). The difference was significant as compared to the dressing (Figure 3J) or Col‐gel (Figure S3H, Supporting Information). To this end, we thoroughly investigated hair follicles neogenesis, as evaluated via co‐immunofluorescence staining of wound tissue samples using various hair follicle‐related markers (alpha smooth muscle actin (α‐SMA), β‐catenin, K10, CD34, K14, and AE15).^[^ ^20^, ^21^, ^22^ ^]^ Upon the co‐stained images of α‐SMA and β‐catenin, nascent hair follicles development was clearly observed in the dermis with FDM‐gel, while highlighted with notably upregulated β‐catenin signals in that region (**Figure** **4**B). We were unable to observe the same phenomenon in the dressing group, in which most of β‐catenin remained in the epidermis (Figure 4A). A Wnt ligand, β‐catenin is a key transducer molecule of Wnt signaling and is required for adult hair follicle growth and regeneration.^[^ ^23^, ^24^, ^25^, ^26^ ^]^ In fact, both Wnt and FGF pathway are known to be deeply involved in hair follicles formation and regeneration of skin wounds in an adult mouse.^[^ ^27^, ^28^, ^29^ ^]^ The cell source of new hair follicles is also of great interest. One study suggested that myofibroblasts reprogramming is required for neogenic hair follicles.^[30]^ Either epidermis‐derived cells or dermal papilla stem cells are also reported to be another candidates.^[^ ^31^, ^32^ ^]^ In a normal skin tissue, highly dense and aligned β‐catenin (+) signals were clearly observed through the epidermis layer (Figure 4C). Another co‐stained images of K10 and CD34 exhibited a significantly different pattern in the epidermis and dermis region. We noticed intense signals of K10 in the epidermis but few CD34 (+) cells in the dermis when treated with dressing (Figure 4D). In contrast, FDM‐gel treated wounds disclosed a wide and populous distribution of CD34, a marker of hair follicle stem cells in the dermis (Figure 4E). It was also interesting to see the distribution pattern of K10 in the FDM‐gel treated wounds, where it was detected through the epidermis and dermis region, which was sharply contrasted with dressing and normal skin as well. For normal skin tissue, K10 was exclusively found in the normal epidermis layer, while CD34(+) cells were present mostly in the dermis but some in the epidermis region (Figure 4F). We also learned a significant difference of hair follicle markers expression in the dermis. As a mature hair follicle was identified via co‐expression of α‐SMA and β‐catenin, we found none in the dressing group, a few from FDM‐gel treated wounds and many in the normal dermis (Figure 4G–I). Another co‐staining of K14 and AE15 showed mature hair follicles in the normal dermis, expressing AE15, which is primarily distributed in inner root sheath cells of human hair follicles (Figure 4L). A few AE15 (+) cells were detected after FDM‐gel treatment (Figure 4K) but none from the dressing (Figure 4J). The cross‐sectional view of the regenerated wound tissue nicely illustrated both sebaceous gland and hair follicles when treated with FDM‐gel (Figure S5A, Supporting Information). Although it was hard to pinpoint the cell source of new hair follicles in this study, we cautiously postulated that as the wound healed, migrating epidermis‐origin cells into the wound bed can be a major cell source. In fact, we noticed that β‐catenin (+) cells were originally located in the epidermis and they were dragged down to the dermis region, producing the neogenic follicles. Moreover, co‐staining of cell proliferation marker Ki67, along with epidermis marker K10 disclosed actively proliferating cells in the wound area (Figure S5B, Supporting Information). Interestingly, some of these cells (marked in arrows) appeared to recapitulate the natural hair morphogenesis stages, such as placode, hair germ, and hair peg.^[^ ^24^, ^33^ ^]^ This result strongly hinted that some of the hair follicles could be newly formed, not originated from the wound edges. It is mentionable, however that current endpoint (2 week) needs to be more extended in the future study to fully assess the regenerative capability of FDM‐gel in wound healing and hair follicles formation. Upon excellent hair follicles formation when administered with FDM‐gel, we designed a mechanistic study and screened β‐catenin activation using human dermal papilla cells (HDPCs) cultivated on FDM. HDPCs adhesion and growth were fine on both TCP and FDM substrate (Figure S6A, Supporting Information). However, cell proliferation was more effective with FDM (Figure S6B, Supporting Information). HDPCs on FDM also disclosed significantly higher level of β‐catenin activation at 3 day than those on TCP as evaluated by co‐staining of α‐SMA and β‐catenin (Figure 4M). The difference was statistically significant (Figure 4N). On the other hand, cutaneous macrophages are known to be involved in wound‐induced hair follicle regeneration.^[^ ^34^, ^35^ ^]^ Hence, we examined the impact of macrophages‐mediated conditioned media (CM) on HDPCs and found that FDM grown macrophages‐derived CM (FDM‐Mac‐CM) was very effective in elevating phosphorylated Akt (*p*‐Akt) of HDPCs, as assessed via western blot (Figure 4O). Quantitatively analyzed, there was no significant difference of total Akt level among five test groups (Figure 4P). However, we knew a notable difference of *p*‐Akt level, where FDM‐Mac‐CM was significantly better than serum‐free or growth media and as effective as bFGF (700 pg mL^−1^) or TCP‐Mac‐CM in upregulating *p*‐Akt (Figure 4Q). Upregulation of *p*‐Akt was closely related to increased HDPCs proliferation.^[^ ^32^, ^36^ ^]^ Aforementioned treatments did not affect HDPCs morphology, cell attachment and growth (Figure S6C, Supporting Information). Further experiment of downstream pathway of Akt exhibited little difference of *p*‐GSK‐3β level when treated with those Mac‐CMs or bFGF (Figure S6D–F, Supporting Information). Our results hinted that macrophage‐mediated paracrine factors could stimulate Akt signaling pathway and as a result, partly contribute to hair follicle formation.

### 2.5 FDM‐Gel Provided Anti‐Inflammatory and Pro‐Healing Milieu During Wound Healing

**Figure 5:**
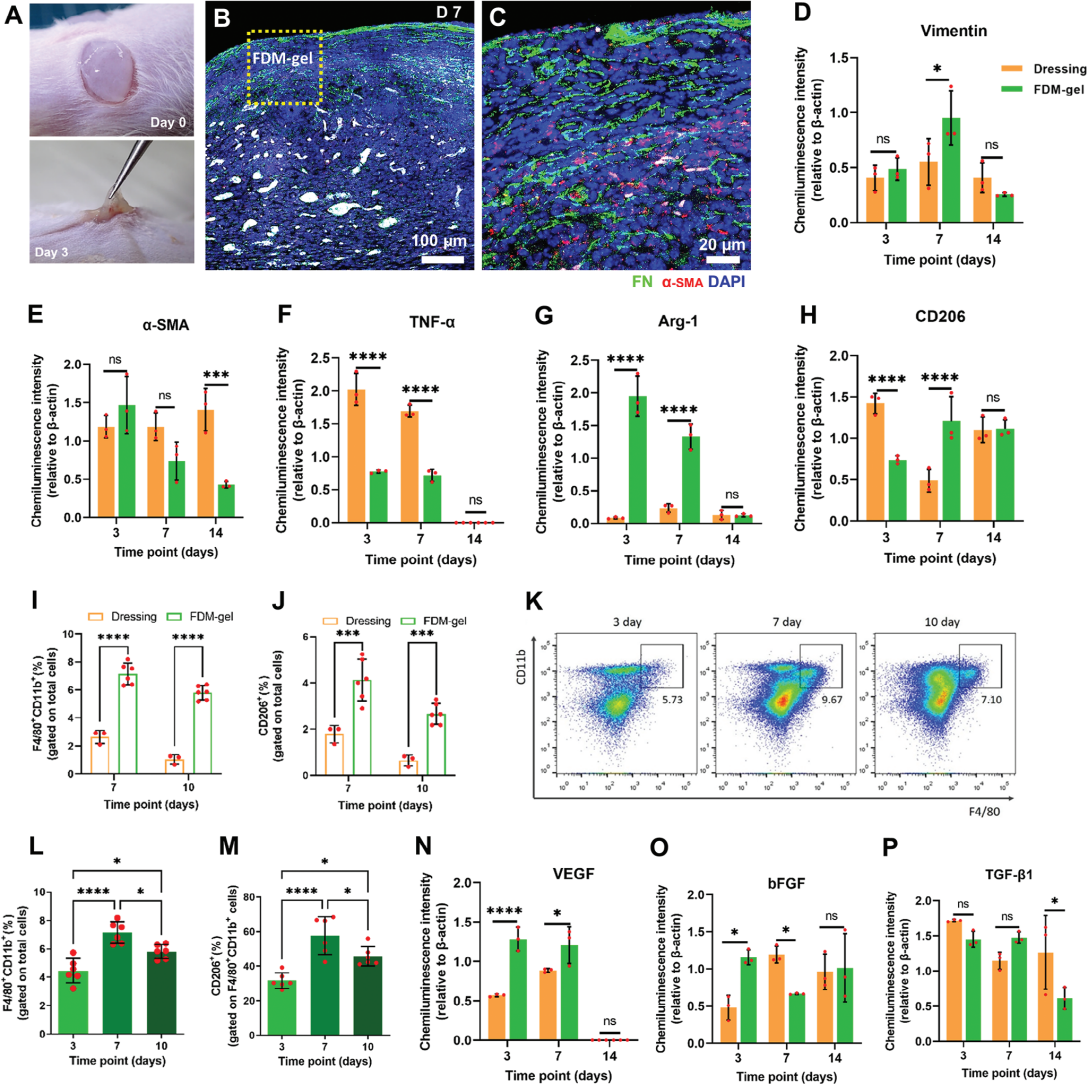
In‐depth analysis of the regenerated wound tissues harvested at specific time points. A) Appearance of FDM‐gel when transplanted into the full‐thickness wound at 0 and 3 day, post‐transplantation. B) Co‐immunofluorescence (FN and α‐SMA) of the FDM‐gel treated wounds at 7 day, along with DAPI staining (Part of FDM‐gel treated area was captured in the yellow box). C) Enlarged image of FDM‐gel treated region in the yellow box. Wound healing associated key proteins and markers were assessed using the regenerating wound tissues via western blot and the comparative data were quantitatively presented at 3, 7, and 14 day, respectively. A fibroblast and myofibroblast marker, D) vimentin and E) α‐SMA. F) Pro‐inflammatory cytokine, TNF‐α. An M2‐like macrophage marker, G) Arg‐1 and H) CD206. FCAS analysis of macrophages population in the wound tissues: I) CD11b^+^/F4/80^+^ cells (%) and J) CD206^+^ cells (%) out of total cells population, as assessed using dressing or FDM‐gel treated wound samples harvested at 7 and 10 day, respectively. K) Representative FACS dot plots for CD11b^+^/F4/80^+^ cells population in the FDM‐gel treated wounds collected at 3, 7, and 10 day, respectively. L) Percentage of CD11b^+^/F4/80^+^ cells out of total cells population as assessed using FDM‐gel treated wound tissues at 3, 7, and 10 day. M) Percentage of CD206^+^ cells among the CD11b^+^/F4/80^+^ cells population. Investigation of the major growth factors detected in the regenerated tissues with time: N) VEGF, O) bFGF, and P) TGF‐β1. Statistically significant difference: *p<0.05, ***p<0.001, or ****p<0.0001.

**Figure 6:**
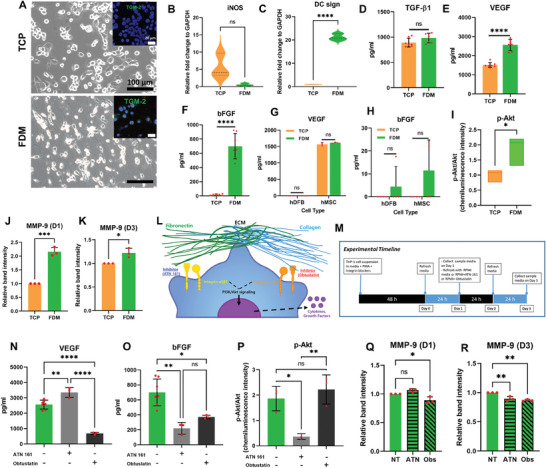
Interactions of macrophage‐FDM via cell surface integrins and their inhibition using specific antagonists. A) THP‐1 cells‐derived macrophages attached to either TCP or FDM. (Inset: An M2‐like macrophage marker, transglutaminase (TGM)−2 positively stained in green). Gene expression of M1‐like macrophage marker, B) iNOS and M2 marker, C) DC sign. Measurement of the growth factors contained in the conditioned media, where the macrophages were growing on two different substrates: D) TGF‐β1, E) VEGF, and F) bFGF. Quantitative analysis of growth factors released from different cell types (human dermal fibroblast‐hDFB and human mesenchymal stromal cell‐hMSC): G) VEGF and H) bFGF. I) An intracellular signaling molecule, *p*‐Akt as assessed by western blot. The MMP‐9 activity of macrophages in vitro when directly interacted with FDM or TCP at J) 1 day and K) 3 day, as evaluated via zymography. L) A schematic illustrates macrophage‐FDM interactions through cell surface integrins (α5β1, α1β1) and specific inhibitors (ATN 161 against α5β1, Obtustatin against α1β1) of such interaction. M) It shows the experimental timeline and the details regarding the macrophage‐ECM interaction and disruption of such interaction using specific integrins inhibitors. Inhibition of specific interaction of macrophage‐FDM and the effect on the secretion of N) VEGF and O) bFGF, as compared to that of non‐treated group (‐/‐). P) Quantitative analysis of *p*‐Akt as assessed via western blot. Measurement of the MMP‐9 activity in the macrophages, when they interacted with FDM (no treatment) or such interaction was inhibited by ATN 161 or Obtustatin at Q) 1 day and R) 3 day, as assessed via zymography. Statistically significant difference: *p<0.05, **p<0.01, ***p<0.001, or ****p<0.0001.

When our FDM‐gel was administered into the full‐thickness wounds, it was conformable and adhesive to the wound area (**Figure** **5**A). The FDM‐gel treated wound tissues (day 7) showed that the transplanted FDM‐gel (fibronectin‐positive) was filled with many host cells (DAPI positive) (Figure 5B), where among them was presumably α‐SMA(+) myofibroblastic cells (red), suggesting that FDM‐gel allowed cellular infiltration other than the immune cells and thereby interacted with myofibroblastic cells (Figure 5C). Western blot analysis of the wound tissues at different time points released numerous information. For each figure graph, representative western blot data appeared in Figure S7 (Supporting Information). First of all, we found enhanced cellular activity of vimentin (+) and α‐SMA (+) cells at early time points but rapidly diminished later (14 day) in the FDM‐gel group (Figure 5D,E). This trend was sharply contrasted with the dressing, where α‐SMA (+) cells were belatedly populous at 14 day (Figure 5E). This result matched with our immunofluorescence result of vimentin/α‐SMA double (+) cells, where they were dominant in the FDM‐gel treated wounds at 7 day, followed by significant decrease later (Figure 3E). Interestingly, myofibroblast is an activated cell type that is responsible for ECM synthesis and organization to restore tissue integrity.^[37]^ However, prolonged presence of myofibroblast in the wound would result in scarring.^[38]^ These results indicate that FDM‐gel may contribute to positively modulating myofibroblast behavior in a time‐dependent manner. Such regenerated wound tissues also exhibited significantly lower level of tumor necrosis factor alpha (TNF‐α) when treated with FDM‐gel (Figure 5F). This is in good agreement with the previous data, where we observed lower level of TNF‐α concentration and decreasing MPO activity in subcutaneously transplanted FDM‐gel (Figure 2H,I). We discovered that an anti‐inflammatory macrophage marker, Arg‐1 was highly elevated in the FDM‐gel treated wounds on day 3 and 7 (Figure 5G). CD206 was also substantially increased at 7 day (Figure 5H). Furthermore, to address the specific macrophage type, FACS analysis of the wound tissues was conducted following the gating strategy (Figure S8, Supporting Information). FDM‐gel group showed notably higher percentage of macrophages (CD11b^+^/F4/80^+^) and M2‐like phenotype (CD11b^+^/F4/80^+^/CD206^+^) as well at 7 and 10 day as compared to that of dressing group (Figure 5I,J). Specifically focused on the FDM‐gel treated wounds, we found highly increasing macrophage cells (CD11b^+^/F4/80^+^) by 7 day and decreased later at 10 day (Figure 5K,L). Interestingly, a significant increase of M2 macrophage (CD206^+^) was observed, especially at 7 day, where it reached to more than 50% on average out of the total macrophage population and declined later at 10 day (Figure 5M). The peaked CD206^+^cells at 7 day seemed to be a turning point of FDM‐gel treated wound healing process. Our results suggested that FDM‐gel may provide an immunomodulatory microenvironment, in which this could suppress severe and/or persistent inflammatory responses during wound healing process. In particular, such time‐dependent macrophage polarization was a very interesting point, because those CD206^+^ macrophages are regarded as M2‐like tissue repair and remodeling macrophages, and also associated with secretion of growth factors, such as VEGF and bFGF.^[^ ^39^, ^40^ ^]^ The role of growth factors on wound healing is definitely essential. We noticed that FDM‐gel treated wounds had significantly higher level of VEGF at early time points (Figure 5N) and bFGF as well at 3 day (Figure 5O). Since VEGF is a well‐known angiogenic factor,^[41]^ such active production of VEGF can explain higher level of neovascularization in the dermis at 7 day (Figure 3C,F). In addition, upon the report that both VEGF and bFGF are essential in hair follicle growth and regeneration,^[27]^ current results partly explained hair follicle formation as observed in the FDM‐gel treated wounds (Figure 3D and Figure 4E). These growth factors are highly produced by macrophages in the wound.^[^ ^42^, ^43^ ^]^ To this end, we speculated that macrophages were responsible to larger amount of VEGF and bFGF secretion, which was triggered by direct interaction of macrophages with FDM‐gel. In fact, our mechanistic study regarding FDM‐macrophage interaction supported this scenario (**Figure** **6**). Moreover, we found no difference of transforming growth factor beta one (TGF‐β1) level by 7 day but FDM‐gel treated wounds exhibited greatly reduced TGF‐β1 at 14 day as compared to the dressing, which still maintained the early TGF‐β1 level (Figure 5P). The difference was statistically significant. This result was notable, because sustained release of TGF‐β1 would lead to fibrosis and scar formation.^[44]^ Although the interplay of such diverse growth factors is complex and hard to decipher the working mechanism exactly, FDM‐gel may foster an immunomodulatory and/or regenerative environment, where FDM‐gel could initiate and encourage pro‐healing activity of accountable cells during wound healing cascade.

### 2.6 Interaction Between Macrophage and FDM Through Specific Cell Surface Integrins was Crucial for Successful Wound Healing

In this study, we have paid much attention to the interactions between FDM‐gel and macrophage for their impact on wound healing. It is natural to imagine that macrophages recognize FDM‐gel as a foreign body and took immediate actions that direct the fate of not only FDM‐gel but wound healing. In fact, macrophages have a profound impact through entire wound healing process, including wound angiogenesis via close interactions with endothelial cells, secretion of pro‐angiogenic factors, matrix degradation and remodeling, and wound‐induced hair follicle regeneration.^[^ ^4^, ^6^ ^]^ We thus recapitulated the FDM‐macrophage interactions in vitro and thoroughly investigated cellular responses. THP‐1 derived macrophages showed a good attachment to both TCP and FDM substrate (Figure 6A). The macrophages on the FDM expressed an M2 marker, tissue transglutaminase‐2 (TGM‐2) (green) but it was barely observed on TCP as assessed via immunofluorescence (Figure 6A, Inset). This was further supported by downregulated expression of pro‐inflammatory marker, iNOS (Figure 6B) and by significantly upregulated anti‐inflammatory DC sign on FDM (Figure 6C). Growth factors secretion by macrophages disclosed that TGF‐β1 was detected in both TCP and FDM but there was no significant difference (Figure 6D). However, we did observe significantly increased VEGF and bFGF level when macrophages were cultivated on FDM as compared to TCP (Figure 6E,F). Upon the previous reports, our result is closely linked to the fact that anti‐inflammatory phenotype macrophages would actively produce pro‐healing growth factors, such as VEGF and bFGF.^[^ ^45^, ^46^ ^]^ Therefore, significantly elevated level of VEGF and bFGF when interacted with FDM pose a significant implication in promoting regenerative wound healing. Interestingly, these results were specific to the FDM as they failed to show increased VEGF or bFGF on the different substrate, such as fibronectin or collagen (Figure S9A,B, Supporting Information). Further investigation using different cell types (humand dermal fibroblast (hDFB) and human mesenchymal stem cell (hMSC)) presented no statistically significant difference between TCP and FDM in the production of VEGF and bFGF (Figure 6G,H). More analysis revealed that FDM could facilitate the phosphorylation of Akt in the macrophages (Figure 6I). The increased *p*‐Akt was indeed the result of upregulated phosphorylation, not the general increase of Akt itself (Figure S9C, Supporting Information), as assessed by western blot (Figure S9D, Supporting Information). Increased phosphorylation of Akt is related to various cellular activities including production of growth factors.^[47]^ Therefore we postulated that FDM‐gel contributed to promoting phosphorylation of Akt in macrophages and this was followed by increased VEGF and bFGF secretion. We hypothesized a mechanism, where enhanced growth factors secretion may be the result of macrophage‐FDM interactions through specific cell surface integrins (Figure 6L). To prove our hypothesis, we treated macrophages using specific integrin blocker, ATN 161 against integrin α5β1 and obtustatin against integrin α1β1, respectively and then allowed cell attachment to the FDM for 24 h, followed by fresh media change, then 24 h incubation before sample collection (Figure 6M). Such treatment did not have any adverse effect on cell survival, proliferation, or attachment to FDM (Figure S10A, Supporting Information). Optimization of integrin blocking treatments suggested that even at very low concentrations, those inhibitors could affect macrophage‐mediated growth factor secretion (Figure S10B–E, Supporting Information). We noticed that VEGF release was affected by only obtustatin, presenting significantly reduced VEGF level (Figure 6N). ATN 161 rather stimulated VEGF secretion. Both ATN 161 and obtustatin, however, had a huge impact in greatly decreasing bFGF secretion as compared to that of no treatment group (Figure 6O). Meanwhile, we also examined such effect using two different substrates, fibronectin and collagen. Both disclosed mostly reduced VEGF or bFGF level, with the integrin blockers treatment (Figure S11A,B, Supporting Information). These results suggested the effect of integrin blockers was not specific to FDM‐gel. Through western blot analysis, we also discovered that the Akt level was barely influenced by inhibitor treatments (Figure S11C, Supporting Information) but the ATN 161‐treated macrophages showed significantly downregulated *p*‐Akt level (Figure 6P), as assessed by western blot data (Figure S11D, Supporting Information). Previous study have shown that interactions between cell and ECM through integrin can activate signaling pathways that lead to increased *p*‐Akt and subsequent growth factor production.^[^ ^47^, ^48^ ^]^ The results of Akt and *p*‐Akt delivered a solid message that macrophage‐FDM interaction through α5β1 can be one of the key events of macrophage response, because *p*‐Akt level was significantly down‐regulated with α5β1 blocking (Figure 6P). Although we were unable to identify the entire downstream signaling pathway, we claim that specific interaction of macrophage integrins and FDM should be a crucial step in initiating the regenerative cascades during wound healing. On the other hand, when matrix remodeling‐related matrix metalloproteinaise (MMP) production was evaluated using zymography, the data disclosed significantly higher MMP‐9 activity when macrophages interacted with FDM than those grown on TCP at 1 and 3 day (Figure 6J,K). There was little change of MMP‐2 activity on both substrates (Figure S9E, Supporting Information). Integrin blocking also affected MMP‐9 activity of macrophages. According to the representative images of the zymography gels (Figure S11E,F, Supporting Information), the blocking of integrin α1β1 (Obs treated) resulted in significantly decreased MMP‐9 production on day 1 but little impact with the integrin α5β1 interruption (Figure 6Q). Interestingly, later time point (day 3) presented a heavy suppression of MMP‐9 activity via blocking of integrin α1β1 and integrin α5β1, respectively (Figure 6R). Integrin blocking rarely influenced MMP‐2 production (Figure S11G,H, Supporting Information) nor did it affect MMP production when macrophages were cultivated on either fibronectin or collagen substrate (Figure S11I, Supporting Information). We acknowledge that matrix remodeling is a critical event for complete tissue regeneration. ECM remodeling occurs at later stage of wound healing, involving many different MMPs derived from different cell sources.^[49]^ MMP‐9 is expressed in several injured epithelia, while contributing to wound healing and cell signaling.^[50]^ MMP‐9 is also produced at the leading edges of migrating keratinocytes during wound closure. Taken together, the increased MMP‐9 could explain the way FDM‐gel was degraded in vivo. MMP‐9 produced by macrophages would break down the ECM, which allowed ECM fragments uptake by macrophages, then resulted in an anti‐inflammatory phenotypes that secreted growth factors.^[51]^ We learned that biodegradation of FDM‐gel via immune cells‐mediated MMPs is another crucial step, which leads to matrix remodeling and eventually wound tissue regeneration.^[52]^

### 2.7 Macrophages Depletion In Vivo Significantly Attenuated the Healing Effect of FDM‐Gel

**Figure 7:**
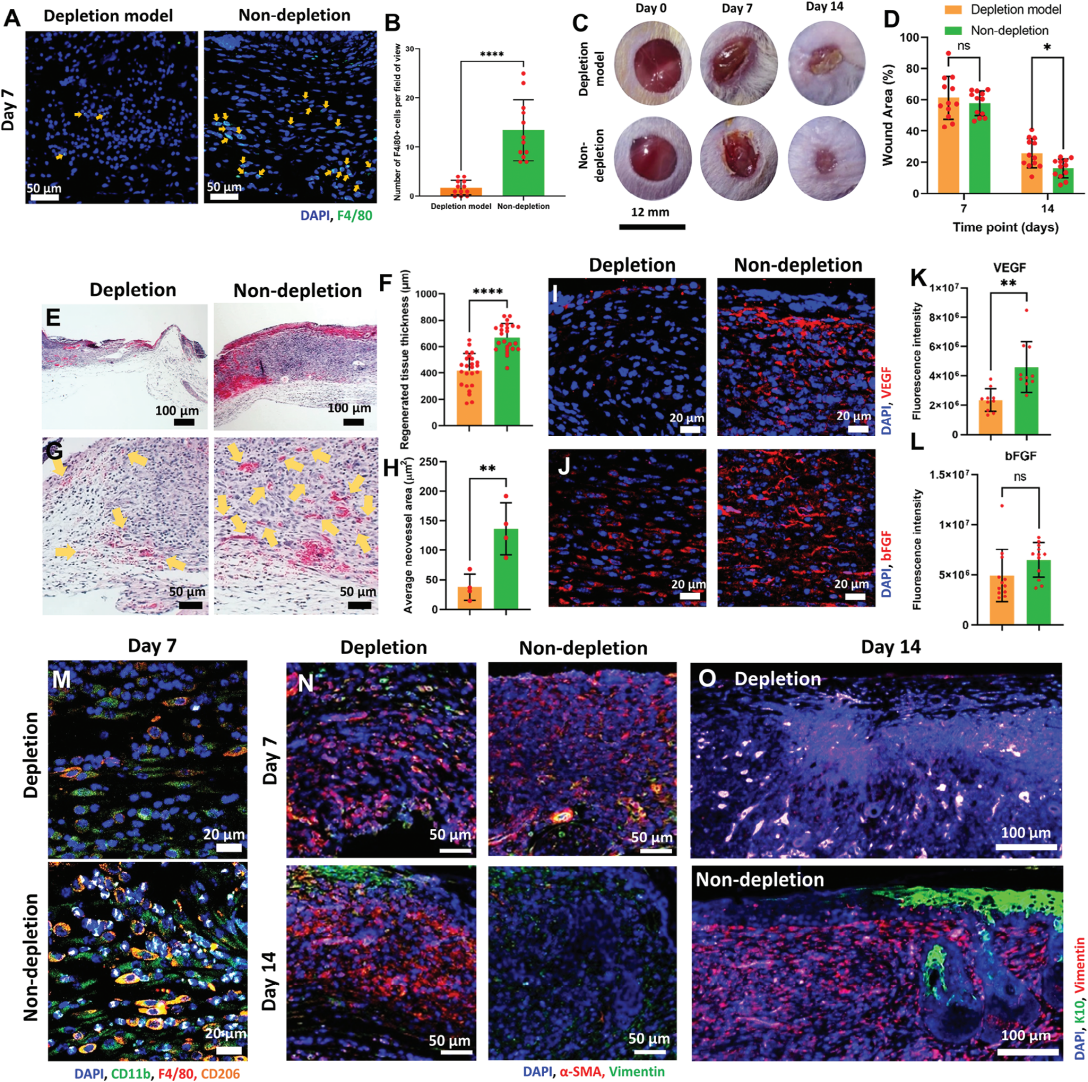
Assessment of inhibitory effect of macrophage‐FDM interaction via macrophage depletion model in vivo. Representative immunofluorescence images of A) F4/80+ cells and B) their quantitative comparison (*n* = 4, each group). C) Gross images of the wounds treated with FDM‐gel in depletion and non‐depletion model at 7 and 14 day, respectively. D) Quantification of the remaining wound area (%) with time. E) Representative images of H&E staining at 7 day. F) Measurement of the wound tissue thickness (µm). G) Histological examination of the wound regions (epidermis and dermis) via H&E staining (Yellow triangles indicate new blood vessels). H) Assessment of the neovessel area (µm^2^) at 7 day. Immunofluorescence of the growth factors, I) VEGF and J) bFGF at 7 day. Quantitative comparison of the growth factors, K) VEGF and L) bFGF as determined using the immunofluorescence images (*n* = 3, each group). M) Co‐immunofluorescence staining of the wound tissues by immune cell markers, CD11b, F4/80, and CD206, along with DAPI staining at 7 day. N) Co‐immunofluorescence of the wound area via vimentin and α‐SMA, along with DAPI staining at 7 and 14 day. O) Analysis of keratinocyte distribution in the epidermis region via K10 staining at 14 day. Statistically significant difference: *p<0.05, **p<0.01 or ****p<0.0001.

Our findings regarding the macrophage and FDM interactions drove us to investigate them further using an in vivo setting. Therefore, we prepared a macrophage depletion model using balb/c mice by the administration of clodronate liposome, which is a clodronate encapsulated liposome.^[53]^ When we first evaluated the wound closure as administered with either clodronate liposome or control liposome without the FDM‐gel treatment, there was no significant difference in wound closure rate between the two groups (Figure S12A,B, Supporting Information). However, the histological analysis disclosed a very poor wound healing as observed in the clodronate‐treated group while liposome group showed a normal healing (Figure S12C, Supporting Information). Further analysis of the macrophage population in the wound disclosed a significant decrease of the macrophages (F4/80+) in the depletion model as compared to the non‐depletion one, thereby confirming successful macrophage depletion (**Figure** **7**A,B). Next, when we equally treated the depletion and non‐depletion wounds using FDM‐gels, we were unable to find any difference in wound closure between them at early time point (Figure 7C). This result was somewhat unexpected one, while previous studies have shown that macrophage depletion can delay the wound healing rate.^[^ ^54^, ^55^ ^]^ The reason may come from different protocols: they administered diphtheria, not clodronate liposome or injected PBS, not liposome vehicle as a control group. Our macrophage depletion was done by subcutaneous injection of clodronate every third day during the whole experiment period (14 days), which possibly depleted only local macrophages and was unable to completely prevent new macrophages migration towards the wound region. On day 14, however, the depletion group showed the remaining wound area (25.9±8.99%), which was substantially larger than that of the non‐depletion one (16.2±5.84%) (Figure 7D). We also noticed a significantly better wound recovery in the non‐depletion group by assessing the thickness of the regenerated tissues at 669.6±102.06 µm, than that of depletion one (419.6±126.86 µm) (Figure 7E,F). Moreover, the average neovessel area showed a huge difference between depletion (37.37±19.39 µm^2^) and non‐depletion (136.22±38.08 µm^2^) (Figure 7G,H). The growth factors, VEGF and bFGF were also found diminished in the depletion model (Figure 7I,J), where VEGF exhibited a statistically significant difference (Figure 7K) but no difference of bFGF between the two groups (Figure 7L), probably due to the fact that ECM itself could be an exogenous source of bFGF. We reasoned that the underdeveloped blood vessels in the depletion model were closely linked to the reduced VEGF level, thus supporting our result that FDM‐macrophage interaction was of importance in elevating VEGF secretion from the macrophage (Figure 6E), which in turn was responsible for increased neo‐vessels in FDM‐gel treated groups (Figure 3L). Compared to the non‐depletion model, we also learned that the depletion one revealed significantly declined macrophage populations in the wound, especially with CD206^+^ M2‐like macrophages (Figure 7M). Interestingly, the non‐depletion model disclosed a very small fraction of myofibroblast at 14 day over the depletion model, which exhibited still large population of α‐SMA(+) cells all the time points (Figure 7N). Such persistence of myofibroblastic cells might be related to the lack of FDM‐macrophage interactions, because we learned that our FDM‐gel could partly contribute to modulating the balance of fibroblast and myofibroblast (Figure 3E and Figure 5D,E). It was also notable that K10 signal in the epidermis was barely detectable in the depletion model as compared to the non‐depletion one (Figure 7O). It is notable, however that in general, our non‐depletion model treated with FDM‐gel was not as effective in wound healing as shown in Figure. 3. First of all, the protocol was significantly different from what we applied for full‐thickness wound model and therefore the results could not be exactly replicated. Although the mechanism is not fully understood at this time, liposome itself might cause an unfavorable impact during wound healing process. Moreover, repeated injection of liposome during the entire experiment period may also have negatively affected the outcome.

In summary, our FDM‐gel demonstrated excellent wound healing capability. FDM‐gel served as a provisional ECM scaffold, in which it prompted innate immune cells recruitment, which was a crucial event at the beginning of wound healing process. FDM‐gel was fully resorbable over time and also immunomodulatory in vivo, where it suppressed the pro‐inflammatory cytokine release and encouraged the production of pro‐healing growth factors. FDM‐gel closely interacted with cell surface integrins of macrophage, where this interaction not only induced M2‐like macrophage polarization but stimulated the release of VEGF and bFGF from the macrophages. Taken together, our FDM‐gel encouraged tissue regeneration by providing a permissive environment for host cells recruitment, including innate immune cells, where FDM‐gel allowed these cells to cooperatively remodel the given environment and eventually gave rise to complete tissue regeneration. Our future study will add more credits while exploring the long‐term effect of FDM‐gel treatment and elucidating specific mechanisms of how FDM‐gel promotes early skin appendage regeneration.

## 3 Experimental Section

### Human Fibroblasts Culture and Decellularization

Human lung fibroblasts (WI‐38, CCL‐75; ATCC) were cultivated on tissue culture plate (TCP) at the density of 2×104 cells cm^−2^ for 9–11 days in Dulbecco's modified Eagle's medium (DMEM) supplemented with 15% fetal bovine serum (FBS), 100 U mL^−1^ penicillin, and 100 µg mL^−1^ streptomycin under normal culture condition (5% CO_2_, 37 °C). To obtain decellularized fibroblast‐derived matrix (FDM), those cells were rinsed with phosphate buffered saline (PBS) twice and subsequently added with decellularization solution (0.25% Triton‐X 100 and 20 mm NH_4_OH). After the removal of the treated solution, 50 U/ml DNase I (18047‐019; Invitrogen) and 2.5 µL mL^−1^ RNase A (12091‐039; Invitrogen) were added and incubated for 1–2 hr at 37 °C. The decellularized FDM was then rinsed with PBS several times and kept at 4 °C for the future use.

### Fabrication of FDM Hydrogel and Col‐Gel

To fabricate FDM‐gel, the confluent human fibroblasts on the 100 mm diameter plate were subjected to decellularization, followed by collection of the decellularized FDM using a cell scraper, then transfer to individual conical tube (50 mL), and addition of deionized water. Next, the tube was subjected to a high speed centrifugation (3500 rpm) for 8 min. The resultant FDM pellet in the tube was the subsequently frozen at −80 °C overnight, and followed by thawing at 37 °C, where this pellet was turned to FDM‐gel via a physical crosslinking. Eventually, FDM‐gels were carefully taken out using forceps and stored at −20 °C for future use. Meanwhile, Col‐gel was also prepared using TeloCol‐10 type I bovine collagen solution (5226; Advanced BioMatrix), where the pH was adjusted to 7.4 using 1N sodium hydroxide. For gelation, Col‐gels (6 mg mL^−1^) were incubated in the 96‐well plate for 30 min at 37 °C.

*Characterization of FDM‐Gel (1): SEM and FT‐IR*: The internal morphology of FDM‐gel was examined using an optical microscope (Zeiss Axio Vert.A1, Germany). For surface microstructure observation, both FDM‐gel and Col‐gel were lyophilized overnight and then subjected to the analysis via scanning electron microscope (SEM; Phenom G2 Pro Desktop, Eindhoven). The molecular compositions of FDM and FDM‐gel were also separately evaluated using Fourier transform infrared (FT‐IR) spectrophotometer (Nicolet 560, Nicolet Co., Madison, WI). All the FT‐IR spectra were recorded in the wavelength ranges of 1000–4000 cm^−1^, with the resolution of 4.0 cm^−1^ and 16‐times scanning.

*Characterization of FDM‐Gel (2): Rheology and Physical Stability*: The rheological property of FDM‐gel was examined via Anton Paar Rheometer (MCR102; Anton Paar), along with the test of collagen gel (Col‐gel) as a positive control. Col‐gel was prepared using TeloCol‐10 type I bovine collagen solution (5226; Advanced BioMatrix), where the pH was adjusted to 7.4 using 1N sodium hydroxide. For gelation, Col‐gels (6 mg mL^−1^) were incubated in the 96‐well plate for 30 min at 37 °C. The rheometer was equipped with a parallel plate (25 mm dia.) and the sample gap size was 0.25 mm. Both storage (G′) and loss modulus (G″) were measured at 37 °C under 5% shear strain. Additionally, a simple test for physical stability was also carried out using Coomassie blue dye, where FDM‐gel and Col‐gel were dipped in the Coomassie blue solution (200 µL) and incubated to allow dye uptake. The stained gels (*n* = 3, each) were transferred into the new dishes, immersed in the PBS, then maintained at 37 °C for up to 14 days. The physical stability of each gel was visually evaluated by dye intensity and gel morphology with time.

*Characterization of FDM‐Gel (3): Human Angiogenesis Array*: Proteome Profiler human angiogenesis array (ARY007; R&D Systems) was employed to screen angiogenesis‐related factors embedded in the FDM‐gel. Briefly, the nitrocellulose membrane containing 55 angiogenesis‐related antibody dots was blocked with supplied block buffer, then treated with a mixture of FDM‐gel lysate and biotinylated detection antibodies cocktail before overnight incubation at 4 °C. After being rinsed with the wash buffer, streptavidin‐horseradish and chemiluminescent detection reagents were added to the membrane sequentially. Once chemiluminescence was processed via iBright CL1500 imaging system, the positive spots were quantitatively analyzed using iBright analysis software.

*Characterization of FDM‐Gel (4): Proteomic Analysis*: The FDM‐gels (*n* = 6) were dissolved in 100 µL of 8 M urea, respectively and denatured at 95 °C for 5 min. After centrifugation at 13 000 rpm for 5 min, 30 µL supernatant was loaded on SDS‐PAGE gel (4‐20% Mini‐PROTEAN TGX Precast Protein Gel), followed by Coomassie blue staining (CoomassieBrilliant Blue R‐250 Staining Solution, BIO‐RAD), then destained (10% acetic acid, 30% MeOH, 60% DW). Sliced gel was then reduced with 20 µL of 100 mm dithiothreitol at 60 °C for 1hr and alkylated by 20 µL of 200 mm iodoacetamide at room temperature (RT) in the dark condition. After then, tryptic digestion of gels using 10 µL of 0.1 µg µL^−1^ Trypsin and 190 µL of 50 mm ABC buffer (ammonium bicarbonate) was carried out at 37 °C for 16 h. The supernatant was concentrated via SpeedVac (HyperVAC‐VC2200, Labogene) at 2000 rpm for 3 h at 4°C. All tryptic peptides were analyzed using an Orbitrap mass spectrometer (Eclipse model, Thermo Fisher Scientific, San Jose, USA) coupled with an Ultimate 3000 nano‐LC system (Thermo Fisher Scientific, USA). Peptides were dissolved in 30 µL buffer A (0.1% formic acid in DW) and 5 µL sample was injected to the nano electrospray ion source, where the injected samples were loaded into trap column (Acclaim PepMap C18 nano Viper 100A, 75 µmx2 cm, 3 µm, Thermo Fisher Scientific) at a flow rate of 5 µL min^−1^ with 95% buffer A. Peptides were then separated via analytical column (PepMap RSLC C18 ES803A, 2 µm, 75µmx50cm, 100A, USA) by 150 min gradient from 5–90% solvent B (0.1% formic acid in acetonitrile) at a flow rate of 300nL min^−1^, where column temperature was maintained at 50 °C. HeLa protein digest standard (100 ng, cat # 88 328; Thermo Fisher Scientific) was evaluated for quality control before and after the sample injection. The Tribrid orbitrap mass spectrometer was operated in a data‐dependent Top20 scan mode switching between MS and MS2. MS and MS/MS spectra were processed and searched by Proteome Discoverer 2.4 (Thermo Fisher Scientific, USA) based on the Sequest HT algorithm using Swiss‐Prot Protein database (Version 2021_07 from human (http://www.uniprot.org)). Raw data were filtered by the number of unique peptide more than 2. False discovery rate (FDR) at the peptide spectral match (PSM) was set at 1% and peptides only showing FDR lower than 1% were selected for peptide identification. For protein quantification, a label‐free quantitation of identified peptides was applied to the protein annotation. Unique and razor peptides were selected as quantifying peptides and protein abundance was calculated by the sum of all quantifying peptide intensity. Abundances were normalized to total peptide amount and scaled with all sample average as 100. Six biological replicates were obtained for each sample. When three of six replicates were missing, the protein was considered not detected and their abundances were replaced with zero value. Averages and *p*‐values were calculated with a Prism software (GraphPad). DAVID knowledgebase v2022q2 (The Database for Annotation, Visualization, and Integrated Discovery, https://david.ncifcrf.gov) was harnessed for the gene ontology (GO) analysis of such proteins contained in the FDM‐gel. Statistical significance of biological processes and molecular functions was determined with *p*‐value.

### Immunofluorescence Staining

The samples obtained from in vitro study were fixed using 4% *p*‐formaldehyde for 30 min at RT. After being rinsed three times with PBS, they were permeabilized by 0.2% Triton‐X 100 and subsequently blocked by 3% bovine serum albumin (BSA) for 1 h. They were then incubated with primary antibodies overnight at 4 °C, followed by adequate washing, subjected to the addition of secondary antibody for 1 h at RT. Such samples were washed again with PBS and mounted onto microscope cover glasses using vectashield mounting medium, added with 4′, 6‐diamidino‐2‐phenylindole (DAPI) (H1200; Vector Lab) for nucleic labeling. Meanwhile, thin sections of in vivo samples were deparaffinized using xylene, rehydrated in a series of alcohol solutions, then subjected to antigen retrieval by microwave heating in citrate buffer (pH 6). Those samples were then blocked with 1% BSA at RT, and subsequently incubated with primary antibody at 4 °C overnight. After several washing, they were incubated with secondary antibody for 1 h at RT and then counterstained with NucBlue Live ReadyProbes Reagent (R37605; Invtirogen), followed by the mounting process with VECTASHIELD Antifade Mounting Medium (H‐1000; Vector Lab). Finally, the immunofluorescence images were photographed using confocal laser scanning microscope (Carl Zeiss). All the samples were tested in triplicates for each group. The information about primary and secondary antibodies used in immunofluorescence can be found in the supplementary material (Table S1, Supporting Information).

### Western Blot

For target proteins analysis of in vitro and in vivo samples, total proteins were extracted from cells and/or tissues. Our samples were washed twice using PBS 1x, subsequently treated with RIPA buffer with protease and phosphatase inhibitor (ab201119; Abcam), then collected using cell scraper, and followed by centrifugation at 13 000 rpm for 10 min at 4 °C. As the supernatant was removed, the total protein content was measured using BCA assay. Next, the protein samples were diluted with 5x SDS‐PAGE loading buffer (SF2002‐110‐00; Biosesang, Korea) and heated for 5 min at 95 °C. Samples and protein ladders were then loaded in the sodium dodecyl sulfate polyacryladmide (SDS‐PAGE) gels (456‐1083; Biorad) and run for electrophoresis under the condition of 200 V and 0.03A. After the operation, the proteins separated in the gel were transferred to the polyvinylidine fluoride (PVDF) membranes (ISEQ00010; Millipore), where they were then blocked by 5% Difco skim milk (23 100; BD Science) dissolved in tris buffered saline with tween‐20 (TBST; Biosesang) for 1 h, and followed by incubation with primary antibody overnight at 4 °C in a shaker. After sufficient washing with TBST, membranes were stained with secondary antibody for 1 h at RT. Followed by another round of washing, they were incubated with chemiluminescence agent for 3 min in a low light at RT. Each chemiluminescence of target proteins was captured by iBright CL1500 Imaging System (Invitrogen, Thermo Fisher Scientific) and quantified using iBright analysis software. All the samples were tested in triplicates for each group. The primary and secondary antibodies used in western blot are listed in the supplementary material (Table S2, Supporting Information).

### Quantitative Reverse Transcription‐Polymerase Chain Reaction

For the analysis of gene expression, total mRNA was extracted from the cells using QIAzol Lysis reagent (Qiagen), following the manufacturer's instruction. Concentration of the isolated RNA was determined at 260 nm using a NanoDrop ND‐1000 spectrophotometer (Thermo Fisher Scientific). The template RNA (1 µg) was mixed in PCR tubes with 4 µL of SUperScript VILO cDNA Synthesis Kit (11 754 050; Thermo Fisher Scientific) to a total volume of 20 mL. They were then subjected to cDNA synthesis reaction at 45 °C for 60 min and RTase inactivation at 95 °C for 5 min, respectively. The resulting cDNA product was utilized in a polymerase chain reaction using ABI Prism 7000 (Applied Biosystems). The gene expression of specific targets was quantified using the 2^−ΔΔCt^ method. The primers of target genes are as follows. iNOS: *GATCAAAAACTGGGGCAGCG* (forward) and *CCTGGGTCCTCTGGTCAAAC* (reverse); DC sign: *GAACTGCGACTCCATCA* (forward) and *GTTGGGCTCTCCTCTGTTCC* (reverse). GAPDH: *CTCAGTGGTGGACCTGACCT* (forward) and *AAAGGTGGAGGAGTGGGTGT* (reverse).

### Subcutaneous Transplantation of FDM‐Gel

All the animal studies were performed in accordance with the Korea Institute of Science and Technology Animal Care and Use Committee Guidelines (KIST‐2021‐04‐053). The BALB/c mice (male, 7 week‐old) were purchased from Orient Bio in Korea. They were anesthetized by gas inhalation using isoflurane in oxygen prior to surgery. Once the mouse hair was shaved by clipper, the dorsal skin was scrubbed using the gauze soaked with alcohol. A minimal incision was made and FDM‐gels were then inserted into two incision sites, respectively for each mouse (*n* = 4). As a comparison purpose, Col‐gel was also subcutaneously positioned following the same protocol. After the incision area was sutured, Tegaderm Film was wrapped around. Gross appearance of those transplants were taken using digital camera. Those mice were then euthanized by CO_2_ inhalation at 1 and 3 day, respectively and the transplanted FDM‐gels and Col‐gels were carefully harvested for further analysis.

### Subcutaneous FDM‐Gel Analysis

The subcutaneous tissues containing FDM‐gel or Col‐gel were collected and fixed in 10% formalin, embedded in paraffin block, and then sectioned in 6 µm thickness across the specimens. Such thin‐sectioned samples were subjected to H&E and immunofluorescence staining, respectively and they were then observed via optical microscope or confocal microscope. Herovici staining (KTHERPT; StatLab, TX, USA) was also carried out to distinguish the difference between immature and mature collagen. Target proteins were vimentin and α‐SMA for fibroblast/myofibroblast and CD45/ F4/80 for macrophages. Isolated FDM‐gel samples (*n* = 8) were also examined for inflammatory responses as assessed via TNF‐α measurement by enzyme‐linked immunosorbent assay (ELISA) and MPO activity by MPO assay. Subcutaneous FDM‐gel implantation was further investigated through macrophage infiltration inside the FDM‐gel, fibrotic capsule formation and FACS analysis of macrophage populations, respectively. The thickness of fibrous capsule layer, where it interfaced with the host tissue was measured using ImageJ, where high magnification microscopic images (*n* = 4) were selected from FDM‐gel and Col‐gel group, respectively. FACS analysis was also performed using only FDM‐gels, because Col‐gels contained few cells inside. The subcutaneous FDM‐gels were carefully harvested, chopped into tiny pieces, then suspended in Gentle Collagenase/Hyaluronidase (Stemcell Technologies, Vancouver, Canada) diluted in RPMI1640. Once the suspensions were incubated for 1 h at 37 °C, the dissociated cells were washed twice with PBS and subjected to the treatment of diluent red blood cell lysis buffer. After centrifugation, the suspended cells were filtered through a cell strainer, centrifuged again, and resuspended in FACS buffer. To prevent non‐specific bindings, the single‐cell suspensions were pre‐blocked with human anti‐CD16/32 for 20 min at 4 °C and they were then labeled with each antibody for specific cell surface markers. Antibodies used for FACS analysis are as follows: rat monoclonal PE/cyanine7 anti‐mouse CD45 (BioLegend; 103 113), rat monoclonal Pacific Blue anti‐mouse/human CD11b (BioLegend; 101 223), and rat monoclonal PE anti‐mouse F4/80 (BioLegend; 123 109). Fluorescence‐activated cell sorting (FACS) data were collected in MA900 (SONY biotechnology, Japan) and analyzed using FlowJo v5.0 (TriStar) software. In addition, in order to confirm the FACS analysis, immunofluorescence staining of the macrophage markers was carried out (CD45, F4/80) using both FDM‐gel and Col‐gel.

### Excisional Full‐Thickness Skin Wound Model

The BALB/c mice (*n* = 8, each group) were randomly divided into three experimental groups: normal (no wound), dressing (Tegaderm) and FDM‐gel. Col‐gel was also tested in a comparison purpose. Once those mice were anesthetized and skin hair was shaved, full‐thickness wounds (two wounds per mouse) were made using a biopsy punch (6 mm) under sterile surgical condition. When the FDM‐gels were transplanted in the wounded area, all wounds were then covered by Tegaderm Film and coban bandage around the wound. FDM‐gels were administered only on day 0 and maintained without the replacement for 14 days post‐transplantation. The wound closure at specific time points was quantitatively measured using ImageJ as a percentage of the wound area normalized to that of day 0. Those mice were euthanized by CO_2_ inhalation on day 7 and 14, respectively. The wound tissue samples were harvested for further in‐depth analysis, where they were carefully incised using scissors and stored in formalin solution.

### Regenerated Wound Tissues Analysis

The wound tissue samples harvested were fixed, embedded in paraffin block, and sectioned following the same protocol as mentioned previously. To evaluate the wound regeneration, the tissue sections were subjected to H&E, Herovici, and immunofluorescence staining, respectively. The antibodies we selected were vimentin and α‐SMA for fibroblast/ myofibroblast, CD31 for neovascularization, and K10 for epidermis regeneration. Another wound healing parameters, such as epidermal and dermal thickness, number of hair follicles, mature collagen ratio, and neovessel area were quantitatively analyzed using ImageJ, where high resolution images (*n* = 8, each group) were randomly selected. For in‐depth analysis of hair follicles formation at 14 day, the tissue sections were also immuno‐stained using hair follicle‐related antibodies and cell proliferation marker, such as β‐catenin, α‐SMA, K10, CD34, K14, AE15, and Ki67. The same procedure was applied using normal skin tissues as a positive control to assess the level of skin appendages recovery when treated with FDM‐gel. Meanwhile, the regenerated wound tissues were carefully harvested at 3. 7, and 14 day, respectively using 12 mm punch and they were then homogenized in RIPA buffer (89 900; Thermo Fisher Scientific) supplemented with a protease inhibitor (R0278; Sigma‐Aldrich). After centrifugation at 13 000 rpm for 10 min at 4 °C, the supernatant was collected and the total protein content was determined via BCA assay (23 227; Thermo Scientific). Western blot analysis was then performed following the protocol previously mentioned. Target proteins are vimentin and α‐SMA for myofibroblast, TNF‐α for inflammation, Arg‐1 and CD206 for M2‐like macrophage, and VEGF, bFGF and TGF‐β1 as a major growth factor in wound healing. Quantitative analysis of western blot was carried out using imageJ, as the intensity of target proteins were normalized to that of β‐actin. The information about primary and secondary antibodies is summarized in the supplementary material (Tables S1 and S2, Supporting Information). Furthermore, to elucidate a time‐dependent profile of M2‐like macrophage population during wound healing, FACS analysis of the regenerating wound tissues harvested at 3, 7, and 10 day, respectively was carried out following the same protocol as previously mentioned in the Section 3.8. Specific antibodies used are as follows: rat monoclonal PE/cyanine7 anti‐mouse cluster of differentiation 45 (CD45) (BioLegend; 103 113), rat monoclonal Pacific Blue anti‐mouse/human CD11b (BioLegend; 101 223), rat monoclonal PE anti‐mouse F4/80 (BioLegend; 123 109), and rat monoclonal FITC anti‐mouse mannose receptor (CD206) (BioLegend; 141 703).

### HDPC Culture and Macrophage‐Mediated Conditioned Media

HDPC (Cefobio, Seoul, Korea) were cultivated using follicle dermal papilla cell growth medium (C‐26501, Promocell), supplemented with the supplement mix (C‐39625; Promocell) and 10 000 units of penicillin, 10 mg streptomycin, and 25 µg amphotericin B (A5955; Sigma‐Aldrich). Upon 80% confluence, isolated HDPCs were seeded and cultivated on two different substrates, TCP and FDM for 3 and 7 days. They were then subjected to immunofluorescence of α‐SMA (an HDPC marker) and β‐catenin, respectively. The β‐catenin (+) cells, where the positive signal was found overlapped with DAPI in the cell nucleus were quantitatively determined using ImageJ, in which high resolution images (*n* = 5) of each group were randomly selected for analysis. Meanwhile, HDPCs were subcultured to 12‐well plate at 2×105 cells per well for 24 h and the medium was replaced with five different media (serum‐free, growth media, two macrophage‐derived conditioned media (Mac‐CM) and bFGF), respectively. Two Mac‐CMs (TCP‐Mac‐CM and FDM‐Mac‐CM) were obtained from the macrophages grown on either TCP or FDM for 24 h. bFGF(700 pg mL^−1^) served as a positive control. Those HDPCs were cultivated for up to 3 days while treated with each medium twice. Isolated HDPCs were then evaluated, specifically targeting Akt, *p*‐Akt, GSK‐3β, and *p*‐GSK‐3β using western blot. Primary and secondary antibodies used are listed in the Table S2 (Supporting Information). Quantitative analysis of western blot was carried out using imageJ, as the intensity of target proteins was normalized to that of β‐actin.

### THP‐1 Cells Culture and Differentiation into Macrophage

THP‐1 cells, human monocyte‐like cells (ATCC) were seeded on TCP and FDM at 3×10^5^ cells mL^−1^ and cultivated in Roswell Park Memorial Institute (RPMI) media (Gibco), supplemented with 10% FBS, 100 U/mL penicillin, and 100 µg mL^−1^ streptomycin. They were then treated with 50 ng mL^−1^ phorbol 12‐myristate 13‐acetat (PMA, Sigma) for 48 h under normal culture condition. This process allowed cell differentiation into M0‐like macrophages and the macrophages adhesion to each substrate as well. Characteristics of macrophages were evaluated via immunofluorescence staining, as preceded by the fixation of macrophages using 4% paraformaldehyde, followed by permeabilization, blocking, and sequential treatments of primary and secondary antibody, which were rabbit polyclonal anti‐transglutaminase 2 (TGM2) (ab421, Abcam) (1:1000) and Alexa Fluor 488‐conjugated goat anti‐rabbit IgG (A11008; Invitrogen) (1:300). The fluorescence images were taken using confocal laser scanning microscope (Carl Zeiss). In addition, another samples obtained from the macrophages grown on either TCP or FDM were prepared for gene expression analysis using QIAzol Lysis reagent (Qiagen). Target genes were inducible NO synthase (iNOS) for M1‐like macrophages and DC sign for M2‐like macrophages. The primers are as follows: iNOS: *GATCAAAAACTGGGGCAGCG* (forward) and *CCTGGGTCCTCTGGTCAAAC* (reverse); DC sign: *GAACTGCGACTCCATCA* (forward) and *GTTGGGCTCTCCTCTGTTCC* (reverse). GAPDH: *CTCAGTGGTGGACCTGACCT* (forward) and *AAAGGTGGAGGAGTGGGTGT* (reverse).

### Macrophage Responses upon Induction or Inhibition of the FDM‐Macrophage Interaction

After 48 h post‐induction of macrophage differentiation on TCP and FDM, fresh media was added and macrophages were incubated for 24 h. The conditioned media was then collected and analyzed for specific growth factors produced by the macrophages, which were VEGF, bFGF and TGF‐β using human VEGF Quantikine ELISA (DVE00, R&D Systems), human bFGF DuoSet ELISA (DY233, R&D Systems) and human TGF‐β DuoSet ELISA (DY240 R&D Systems), respectively. Different cell types other than macrophages, such as hDFB and hMSC were also assessed for VEGF and bFGF secretion following the same protocol. Moreover, different ECM substrates, collagen or fibronectin‐coated substrate were also prepared and tested for the same purpose. Meanwhile, to investigate an inhibitory effect of the macrophage‐FDM interactions, we seeded THP‐1 cells‐derived macrophages on the FDM and subsequently added the media supplemented with ATN‐161 (SML2079; Sigma‐Aldrich), an integrin α5β1 receptor antagonist, or supplemented with obtustatin (4664; Tocris), an integrin α1β1 receptor antagonist. Different concentrations of such antagonists were also tested: 1, 10, 50, and 100 µmol L^−1^ for ATN‐161 and 50, 100 ng mL^−1^, 1 and 5 µg mL^−1^ for obtustatin. After 48 h, the media were collected and analyzed for VEGF and bFGF content using ELISA. We also prepared different substrates, collagen and fibronectin‐coated one and tested them for the same purpose. Additionally, the macrophages grown on TCP and FDM or those treated with such integrins antagonists were analyzed via western blot, specifically targeting a cell signaling molecule, Akt and *p*‐Akt, respectively. Quantitative analysis of western blot was carried out using imageJ, as the intensity of target proteins was normalized to that of β‐actin. All the samples were tested in triplicates for each group.

### Zymography

To assess MMP activity of macrophages when directly interacted with FDM, THP‐1 were seeded and cultivated on the FDM, with or without integrins antagonist treatment, following the same procedure as described in the section of 3.14. After 48 h, fresh media was replenished and macrophages were incubated for 1 and 3 day, followed by CM collection from four different test groups: TCP for the macrophages grown on TCP, NT for the cells on the FDM without integrins antagonists, ATN for the cells on the FDM treated with ATN 161, and Obs for the cells on the FDM treated with obtustatin. We also added a control group, which is just growth media (GM) without cells. Each CM was mixed with 5x non‐reducing sample buffer. Those samples (TCP, NT, ATN, Obs) were then loaded in the SDS‐PAGE gel containing gelatin. Once electrophoresis was completed, the gels were maintained overnight at 37 °C in the incubation buffer containing zinc and calcium to activate MMPs. Eventually, those gels were subjected to Coomasie blue staining, where MMP activity of each sample was identified as a white band against a dark blue background. Additionally, we also investigated the MMP activity of macrophages cultivated on collagen and fibronectin substrate, respectively. We evaluated MMP‐9 and MMP‐2 activity and quantified each white band using ImageJ.

### Macrophage Depletion Animal Model using Clodronate Liposome

The BALB/c mice (*n* = 3, each group/ two wounds per mouse) were divided into two experimental groups: clodronate liposome‐treated and liposome‐treated one. Prior to the FDM‐gel administration to the full‐thickness wounds, we injected 100 µL of either control liposome or clodronate liposome (Liposoma, Amsterdam, Netherlands) into the mice subcutaneously at the periphery of the wounds. Clodronate liposomes and control liposome were re‐administered every third day to maintain macrophage depletion status in vivo. All the wounds were treated with FDM‐gels and maintained for up to 14 days post‐transplantation. The wound size was quantitatively measured at day 7 and 14 using ImageJ as a percentage of the wound area normalized to that of day 0. The mice were euthanized by CO_2_ inhalation and the wound tissues were harvested for the extensive analysis, including histology and immunofluorescence as described in the previous sections. In addition, to confirm the quality of macrophage depletion model, we prepared full‐thickness wounds and injected the chemicals as mentioned in this section, followed by no treatment of FDM‐gels. To confirm the macrophages depletion, the wounds samples (*n* = 4, each group) were subjected to immunofluorescence staining of the pan‐macrophage marker, F4/80. The number of F4/80+ cells were then determined through manual counting of positive cells in three random field of views.

### Statistics

Statistical analysis was performed via GraphPad Prism 9 software. All the data presented are mean ± standard deviation. Two‐tailed (α = 0.05) student t test was employed for two experimental groups. One‐way ANOVA with a post hoc Tukey's multiple comparison test was carried out for more than three test groups. A statistically significant difference was denoted as *p < 0.05, **p < 0.01, ***p < 0.001, or ****p < 0.0001.

## Conflict of Interest

The authors declare no conflict of interest.

## Supporting information

Supporting Information

## Data Availability

The data that support the findings of this study are available from the corresponding author upon reasonable request.
