# Supplementary material for: Human Fibroblast‐Derived Matrix Hydrogel Accelerates Regenerative Wound Remodeling Through the Interactions with Macrophages
Source: Adv Sci (Weinh). 2024 Mar 12;11(18):2305852. doi: 10.1002/advs.202305852 (PMC11095160; doi:10.1002/advs.202305852)
Supplement: Supplementary file 1 — Supporting Information [file ADVS-11-2305852-s001.pdf]

ADVANCED SCIENCE
Open Access

Supporting Information

for *Adv. Sci.*, DOI 10.1002/advs.202305852

Human Fibroblast-Derived Matrix Hydrogel Accelerates Regenerative Wound Remodeling Through the Interactions with Macrophages

*Cininta Savitri, Sang Su Ha, Jae Won Kwon, Sung Hoon Kim, Young-Min Kim, Hyun Mee Park, Haejin Kwon, Mi Jung Ji and Kwideok Park**

Human fibroblast-derived matrix hydrogel accelerates regenerative wound remodeling through the interactions with macrophages

Cininta Savitri[a,1], Sang Su Ha[a,1], Jae Won Kwon [a,b,1], Sung Hoon Kim [a], Young-Min Kim [a,b], Hyun Mee Park [c], Haejin Kwon [c], Mi Jung Ji [c], and Kwideok Park[a,b,*]

[a] Center for Biomaterials, Korea Institute of Science and Technology (KIST), Seoul 02792, Republic of Korea

[b] Division of Bio-Medical Science and Technology, KIST School, University of Science and Technology (UST), Seoul 02792, Republic of Korea

[c] Advanced Analysis and Data Center, KIST, Seoul 02792, Republic of Korea

[1]Equal contribution: Cininta Savitri, Sang Su Ha, and Jae Won Kwon

Running title: *FDM-gel enables regenerative wound remodeling*

Submitted to *Advanced Science*

*Correspondence: Kwideok Park, Ph.D.

E-mail: kpark@kist.re.kr

Tel: +82-2-958-5288

Fax: +82-2-958-5308

August 2023

Table S1. List of primary and secondary antibody used for immunofluorescence

Primary antibody	Commercial product #	Dilution ratio
Mouse monoclonal anti-fibronectin (FN)	sc-8422	1:200
Rabbit polyclonal anti-fibronectin (FN)	ab2413	1:200
Rabbit polyclonal anti-collagen type I (Col I)	ab34710	1:300
Rabbit polyclonal anti-collagen type III	ab7778	1:100
Mouse monoclonal anti-α-smooth muscle actin (α-SMA)	a2547	1:300
Rabbit polyclonal anti-α-smooth muscle actin (α-SMA)	ab5694	1:100
Mouse monoclonal anti-CD34	sc-7324	1:150
Rabbit polyclonal anti-CD31	ab28364	1:100
Mouse monoclonal anti-CD68	ab955	1:50
Mouse monoclonal anti-vimentin	sc-373717	1:300
Mouse monoclonal anti-pan-cytokeratin (AE13)	sc-57012	1:50
Rabbit monoclonal anti-cytokeratin 10	ab76318	1:500
Rabbit polyclonal anti-β-catenin	ab16051	1:300
Rabbit polyclonal anti-transglutamine 2 (TGM2)	ab421	1:1000
Rat monoclonal anti-F4/80	ab16911	1:200
Rabbit monoclonal anti-CD45	ab282747	1:200
Mouse monoclonal anti-basic fibroblast growth factor (bFGF)	sc-74412	1:200
Rabbit polyclonal anti-VEGFA	ab51745	1:50
Rabbit monoclonal anti Ki67	ab16667	1:50
Mouse monoclonal anti-myosin heavy chain (MHC)	ab37484	1:300
**Secondary antibody**	**Commercial product #**	**Dilution ratios**
Alexa Fluor® 488	A11001	1:300
Alexa Fluor® 594	A21207	1:300
Alexa Fluor® 594	ab1501060	1:200

Table S2. List of primary and secondary antibody used for western blot

Primary antibody	Commercial product #	Dilution ratio
Rabbit polyclonal anti-collagen type I (Col I)	ab34710	1:500
Mouse monoclonal anti-fibronectin (FN)	sc-8422	1:500
Rabbit polyclonal anti-α-smooth muscle actin (α-SMA)	ab5694	1:500
Mouse monoclonal anti-vimentin	sc-373717	1:500
Mouse monoclonal anti-tumor necrosis factor alpha (TNF-α)	ab8348	1:500
Rabbit polyclonal anti-Vascular endothelial growth factor (VEGF)	ab46154	1:1000
Mouse monoclonal anti-basic fibroblast growth factor (bFGF)	sc-74412	1:500
Rabbit monoclonal anti-Phospho-Akt (p-Akt)	244F9	1:1000
Rabbit monoclonal anti-Akt	9272S	1:1000
Mouse monoclonal anti-arginase 1	sc-271430	1:500
Rabbit polyclonal anti-mannose receptor (CD206)	ab64693	1:500
Mouse monoclonal anti-β-actin	SC47778	1:500
Rabbit monoclonal anti-GSK-3β	12456S	1:1000
Rabbit monoclonal anti- Phospho-GSK-3β (p-GSK-3β)	9336S	1:1000
**Secondary antibody**	**Commercial product #**	**Dilution ratio**
Mouse IgG (H+L) HRP	31430	1:200,000
Pierce® goat anti-rabbit IgG (H+L) peroxidase conjugated	31460	1:200,000

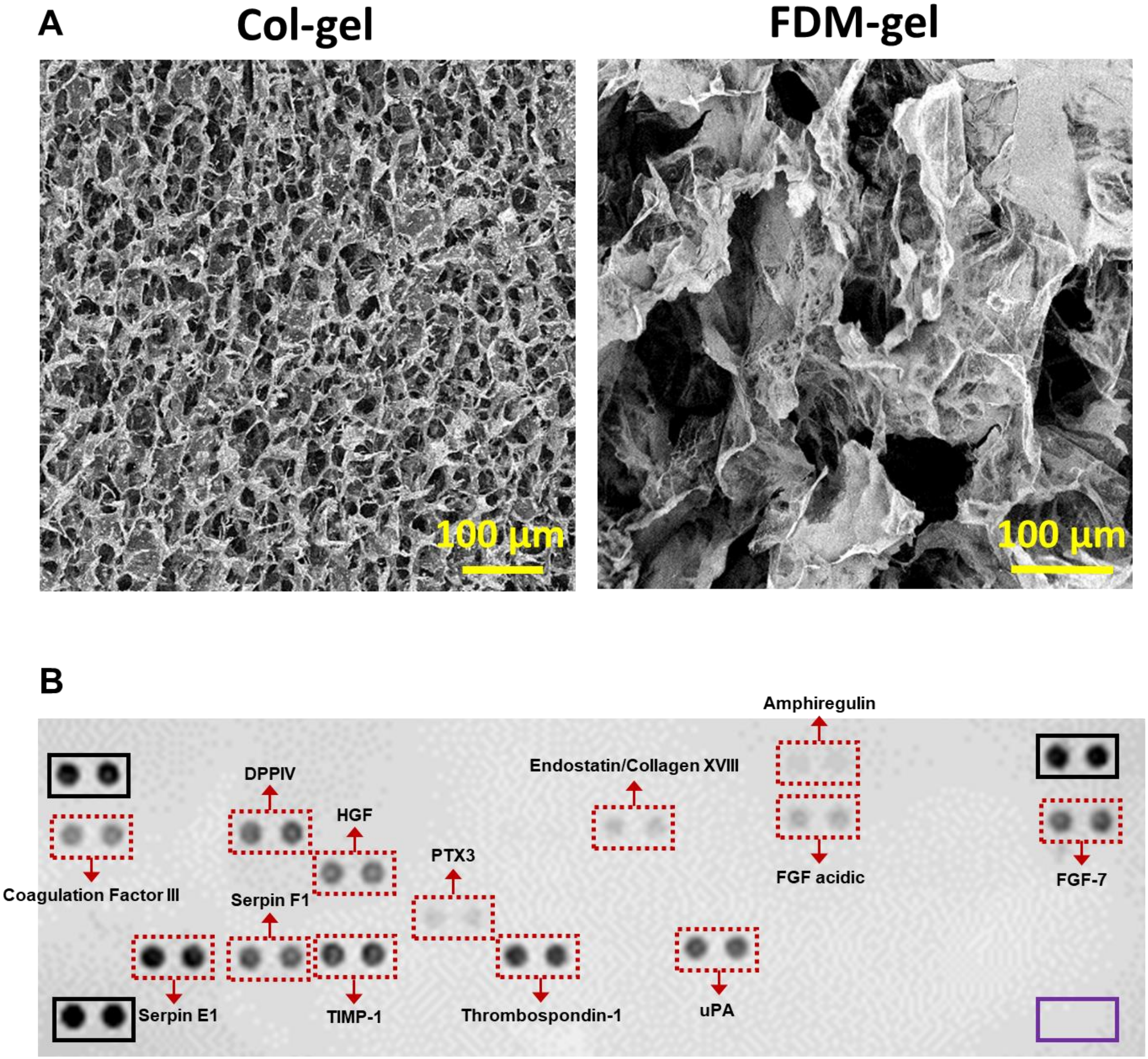

Fig. S1. (A) Cross-section images of Col-gel and FDM-gel, respectively as taken by SEM. (B) Biochemical analysis of FDM-gel. Proteome Profiler™ human angiogenesis array for angiogenic-related factors screening in FDM-gel. Positive controls (3) appear in the two black dots boxed at the corners and negative control (1) appears nothing in the purple colored box. Some notable factors in their intensity are marked in the boxes of red-dotted line.

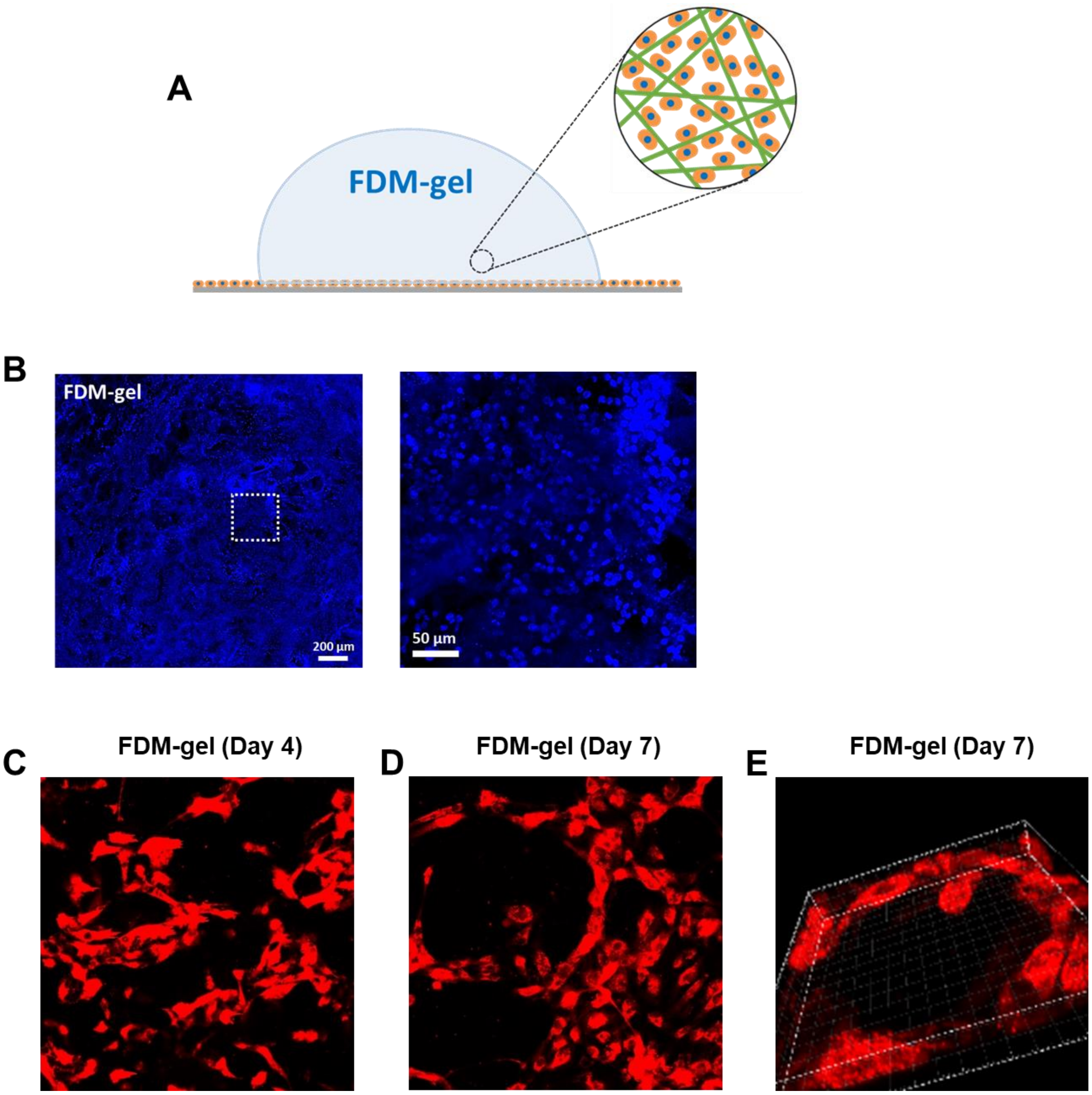

Fig. S2. Examination of angiogenic capability of FDM-gel *in vitro* using the HUVECs encapsulated in the FDM-gel. (A) Schematic of HUVECs-contained FDM gel. (B) Observation of cellular distribution inside the FDM-gel via DAPI staining of HUVECs. (C, D, E) Evaluation of tube formation of HUVECs in the FDM-gel at 4 and 7 day, respectively by using CD31 immunostaining.

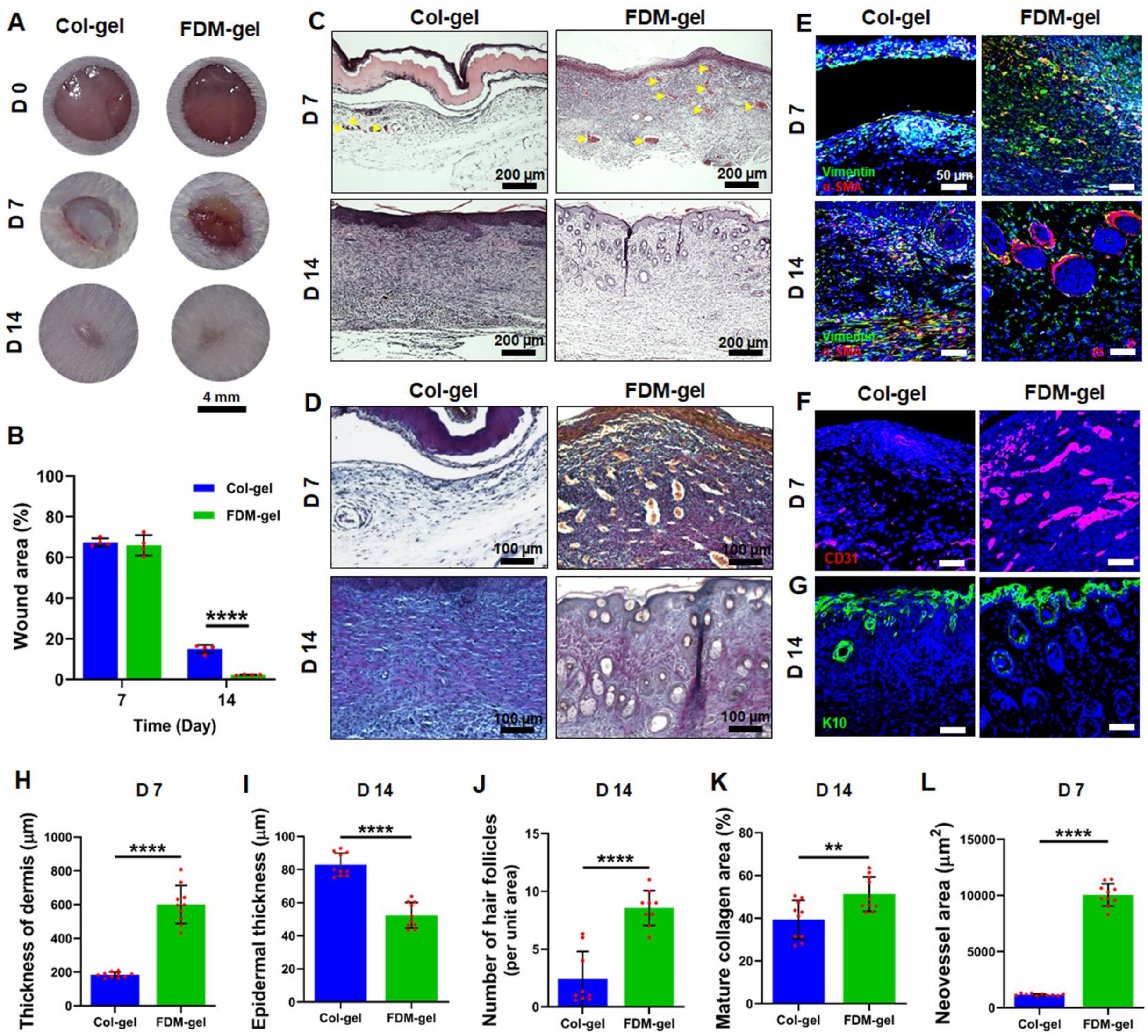

Fig. S3. In vivo transplantation of Col-gel in the full-thickness skin wound model and evaluation of wound healing effect, along with FDM-gel at different time points. (A) Gross appearance of the wounds treated with Col-gel and FDM-gel at 7 and 14 day post-treatment, respectively. (B) Measurement of the wound area (%) after Col-gel or FDM-gel treatment at specific time points. Histological analysis of regenerated wound tissues at 7 and 14 day: (C) H&E and (D) Herovici staining, respectively. (E) Vimentin and α-SMA staining at 7 and 14 day. (F) CD31 staining on day 7. (G) K10 staining at 14 day. Quantitative analysis of wound healing parameters: (H) Thickness of dermis, (I) Epidermal thickness, (J) Number of hair follicles, (K) Mature collagen ratio (%), and (L) Neovessel area. Statistically significant difference: **p<0.01 or ****p<0.0001.

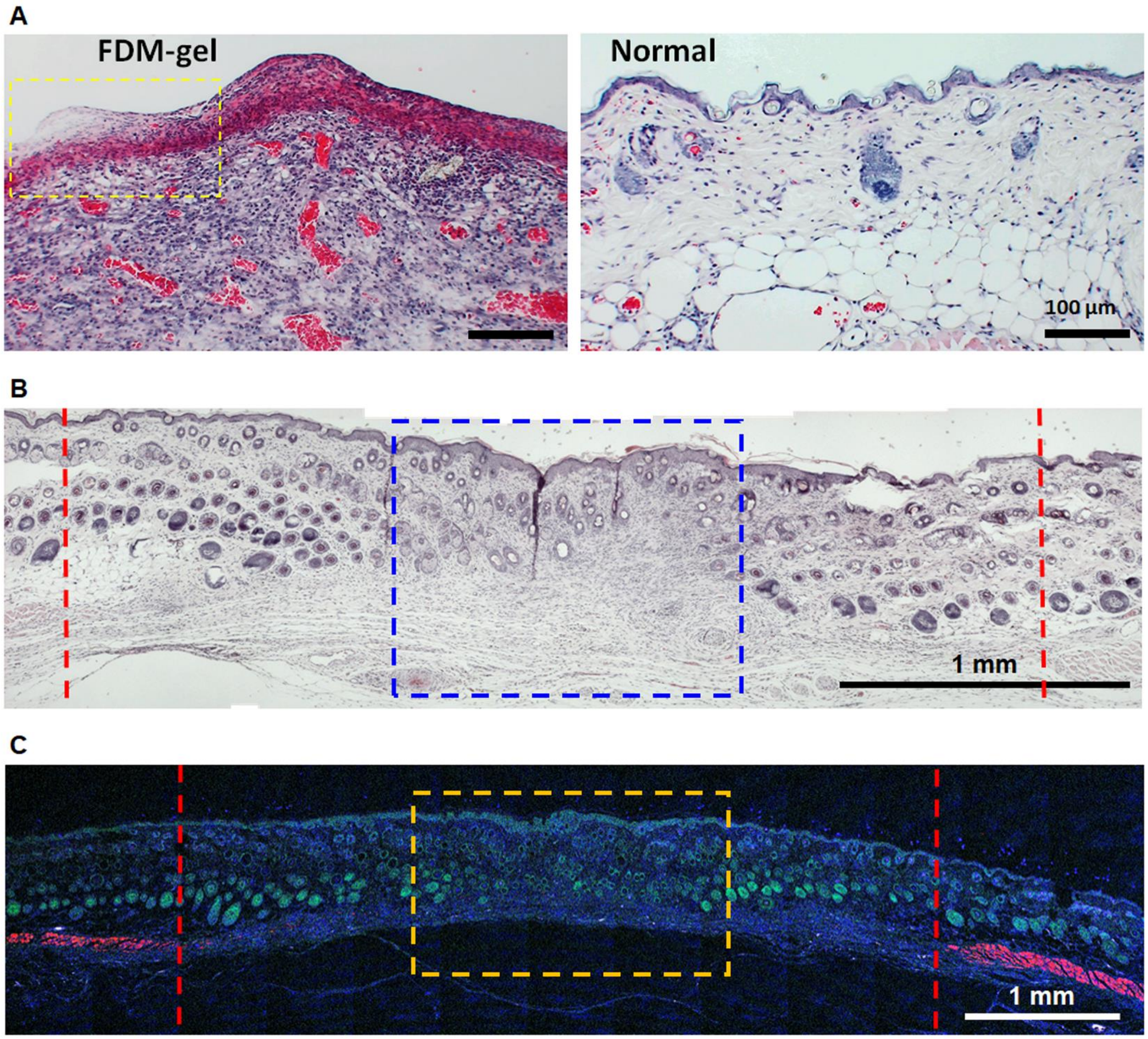

Fig. S4. (A) A high magnification image of FDM-gel treated wound region at 7 day (Fig 3C), where it showed the epidermis was not fully developed compared to the normal tissue. Yellow dotted box region clearly shows underdeveloped epidermis. (B) Representative whole FDM-gel treated wound images on day 14. Red dotted lines mark the wound edges and blue dotted box matches the region as shown in Fig. 3C. C) Immunofluorescence of β-catenin (green) marks the regenerated hair follicles, while myosin heavy chain staining (pink/red) shows the area of panniculus carnosus muscles, not fully regenerated. The orange-color dotted box region is equal to the blue dotted box in the Fig S4A, both of which represent the wound area.

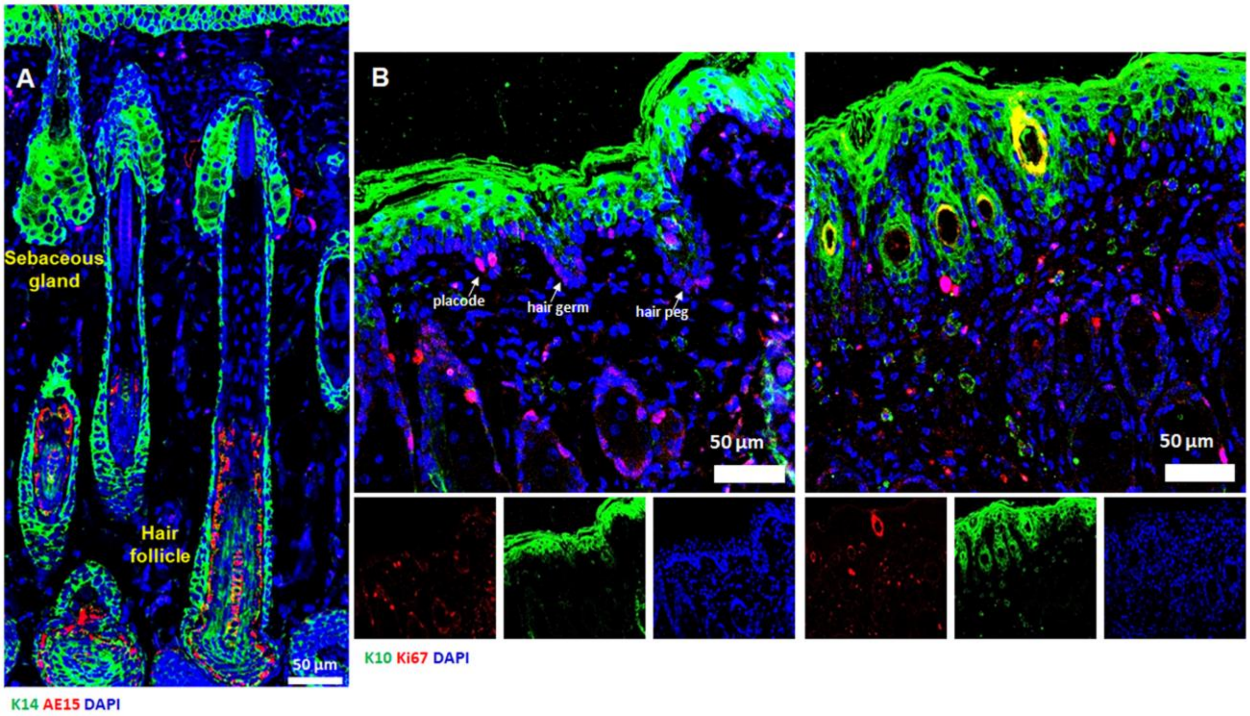

Fig. S5. (A) Representative merged and individual images of Ki67 and K10 staining displayed hair follicles morphogenesis in the wound area at 14 day after the FDM-gel treatment. (B) A representative photograph shows new hair follicles development in the wound area when administered with FDM-gel at 14 day post-treatment. Co-immunofluorescence staining of K14, a structural support in the basal keratinocytes of epidermis and AE15, which is distributed primarily in inner root sheath cells of hair follicles, along with DAPI staining.

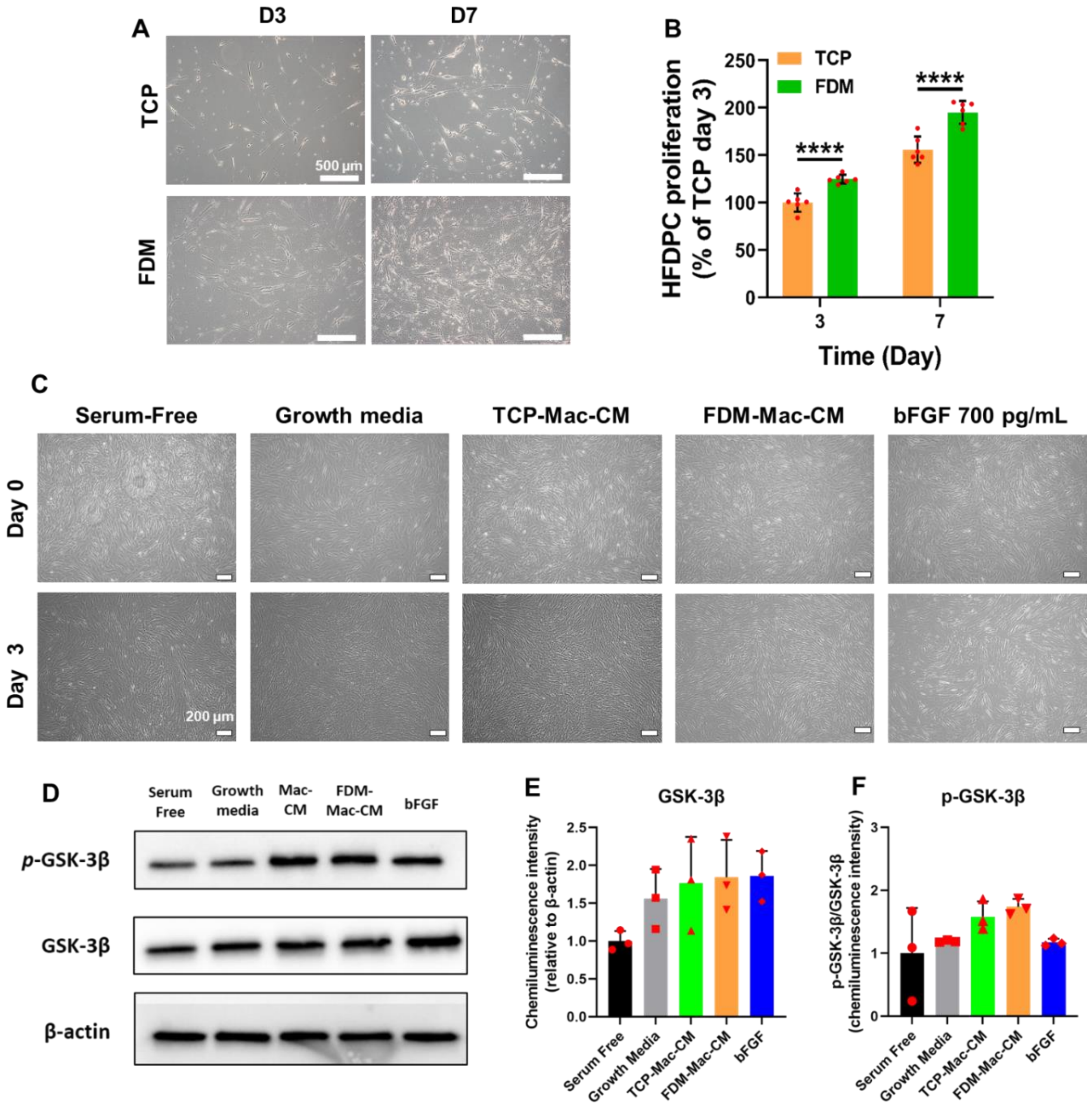

Fig. S6. Investigation of hair follicle dermal papilla cells (HFDPC) responses *in vitro*. (A) HFDPC adhesion and growth on TCP or FDM substrate at 3 and 7 day, respectively. (B) HFDPC proliferation on TCP or FDM for up to 7 days. (C) HFDPC culture for 3 days under five different media conditions: serum-free, growth media, TCP grown macrophage-derived conditioned media (TCP-Mac-CM), FDM grown macrophage derived conditioned media (FDM-Mac-CM), and bFGF (700 pg/mL), respectively. Analysis of downstream pathway of Akt in the HDPCs when treated with five different media formulations. (D) Upon such treatments, both GSK-3β and phosphorylated GSK-3β (*p*-GSK-3β) level of HDPCs were assessed via western blot. Quantitative analysis of (E) GSK-3β and (F) *p*-GSK-3β. Statistically significant difference: ****$p<0.0001$.

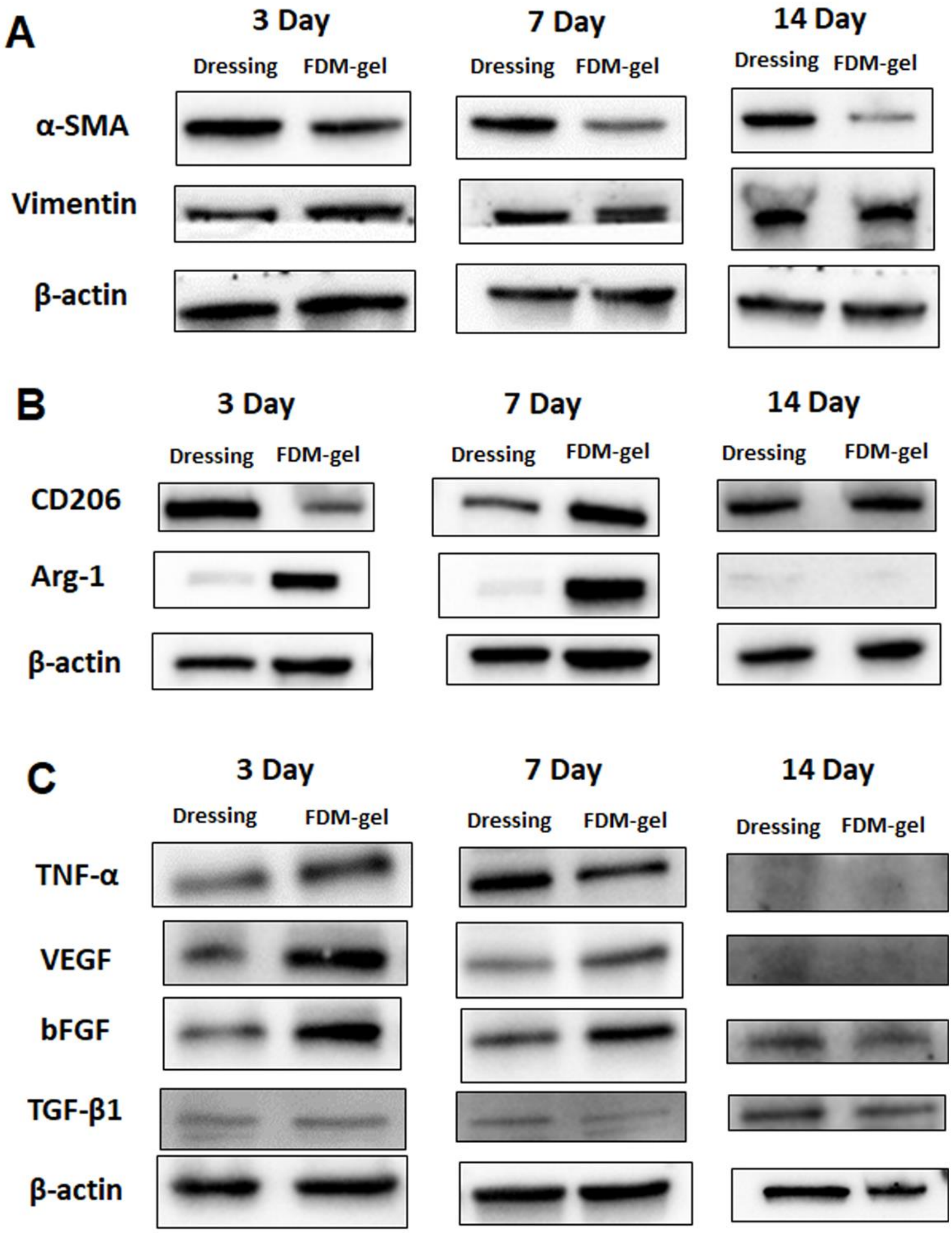

Fig. S7. Representative data of western blot results as evaluated from the regenerated wound tissues at 3, 7, and 14 day, respectively when treated by either dressing or FDM-gel. (A)

Identification of myofibroblastic cell markers (α-SMA and vimentin) in the regenerated wound tissues. (B) M2 macrophage polarization markers (CD206, Arg-1). (C) Growth factors/cytokines level at different time points.

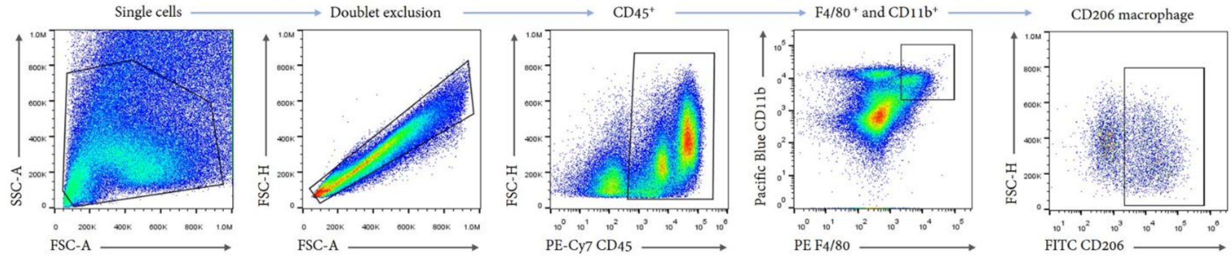

Fig. S8. Gating strategy of flow cytometry analysis as presented in the Figure 5R-V.

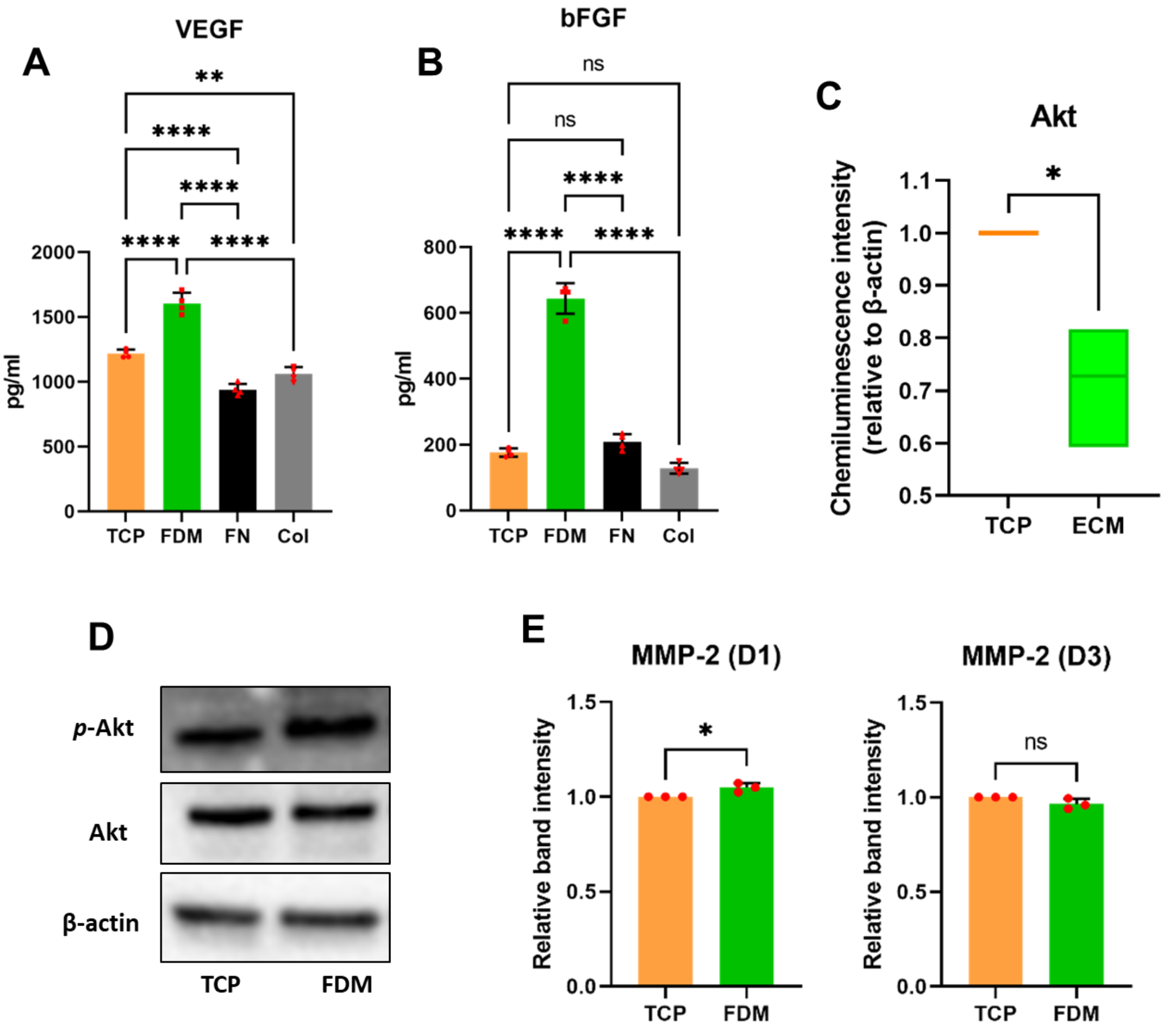

Fig. S9. Analysis of growth factors, Akt/ *p*-Akt, and MMP-2 as assessed from the macrophages *in vitro*, where they grow on different substrates. Measurement of (A) VEGF and (B) bFGF secreted from macrophages cultivated on four different substrates. TCP and FDM, fibronectin (FN), and collagen (Col). (C) Quantification of Akt out of macrophages cultivated on TCP or FDM. (D) Representative western blot data of *p*-Akt and Akt as obtained from macrophages on TCP or FDM. (E) Quantification of MMP-2 secreted by macrophages cultured on either TCP or FDM based on zymography analysis on day 1 and 3. Statistically significant difference: *$p<0.05$, **$p<0.01$ or ****$p<0.0001$.

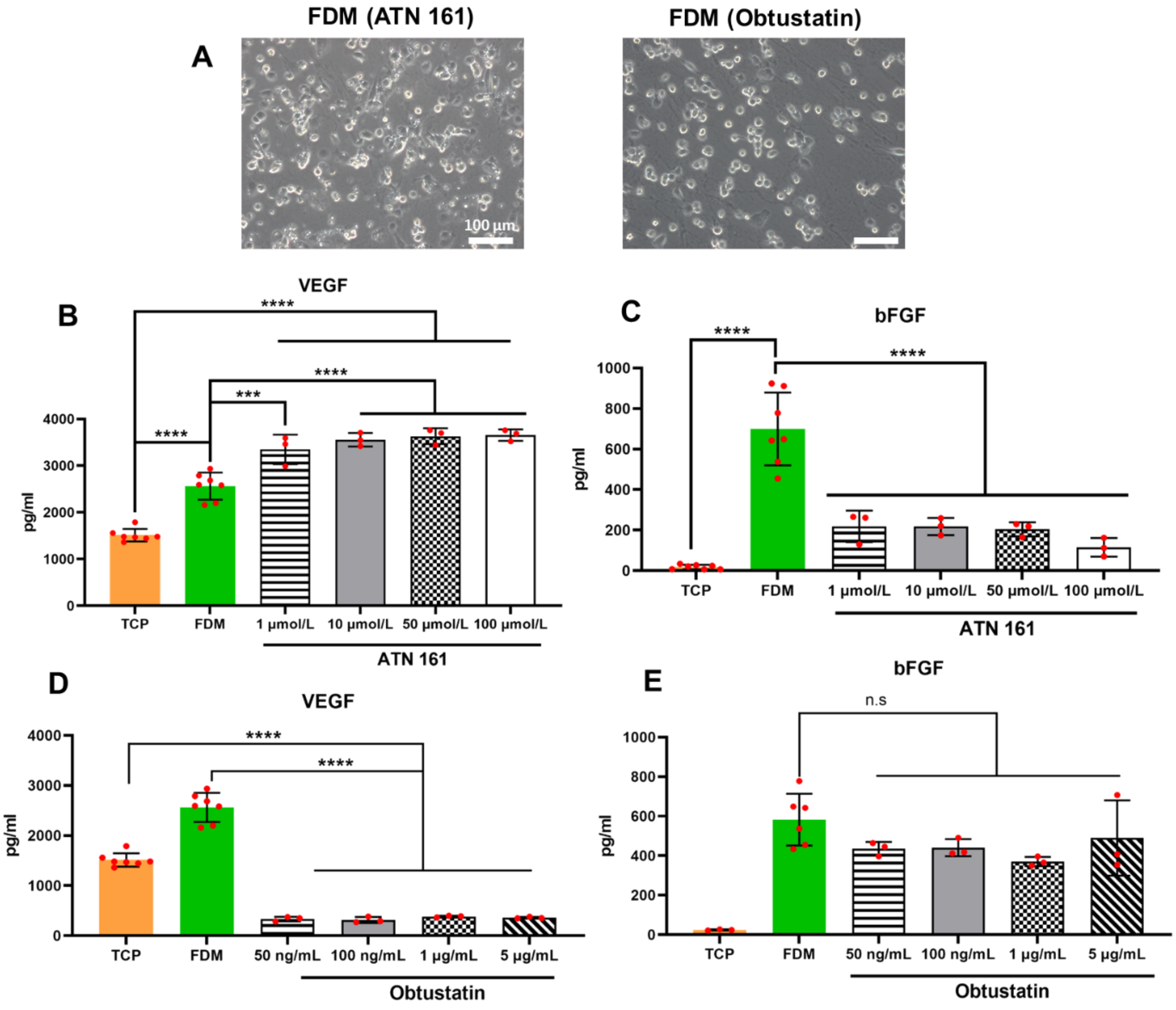

Fig. S10. Treatment of specific integrin blockers in a series of concentrations and their impact on the growth factors release out of the macrophages on FDM. (A) Macrophages attachment and morphology on FDM when treated with either ATN161 (α5β1 inhibitor) or Obtustatin (α1β1 inhibitor). Effect of different concentrations of ATN161 treatment on the secretion of (B) VEGF and (C) bFGF, respectively. Effect of different dosages of Obtustatin on the release of (D) VEGF and (E) bFGF, respectively. Statistically significant difference: ***p<0.001 or ****p<0.0001.

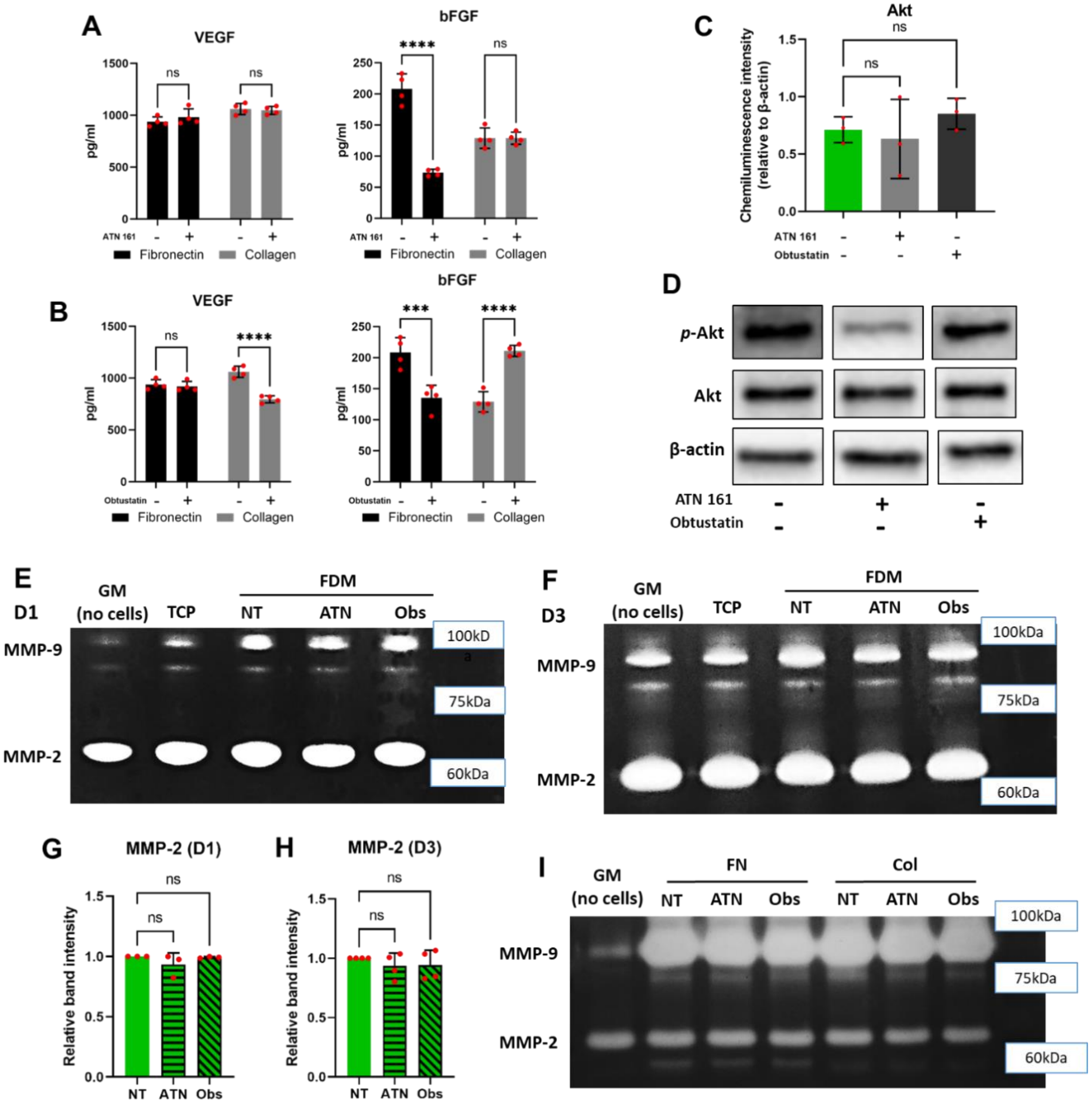

Fig. S11. Effect of integrin blocking on macrophage-substrate interactions. (A) Measurement of VEGF and bFGF secreted from macrophages cultivated on fibronectin (FN) and collagen (Col), respectively with or without the α5β1 integrin blocker, ATN161. (B) Measurement of VEGF and bFGF level on fibronectin (FN), and collagen (Col), respectively with or without the α1β1 integrin blocker, Obtustatin. (C) Assessment of Akt level of the macrophages cultured on FDM, with or without integrin blockers. (D) Representative western blot result for *p*-Akt, Akt, and β-actin from the macrophages cultured on FDM, when they treated with or without integrin blockers.

Representative gel images of zymography for MMP-9 and MMP-2 on (E) day 1 and (F) day 3, where macrophages on FDM were examined by treating two different integrin blockers, along with macrophages on TCP or growth media (GM) alone as a control group. (G, H) Quantification of MMP-2 level out of the zymography results at different time points (E and F). (I) Representative gel images of zymography from the macrophages on either FN or Col substrate, with or without integrin blocking. Statistically significant difference: ***$p<0.001$ or ****$p<0.0001$.

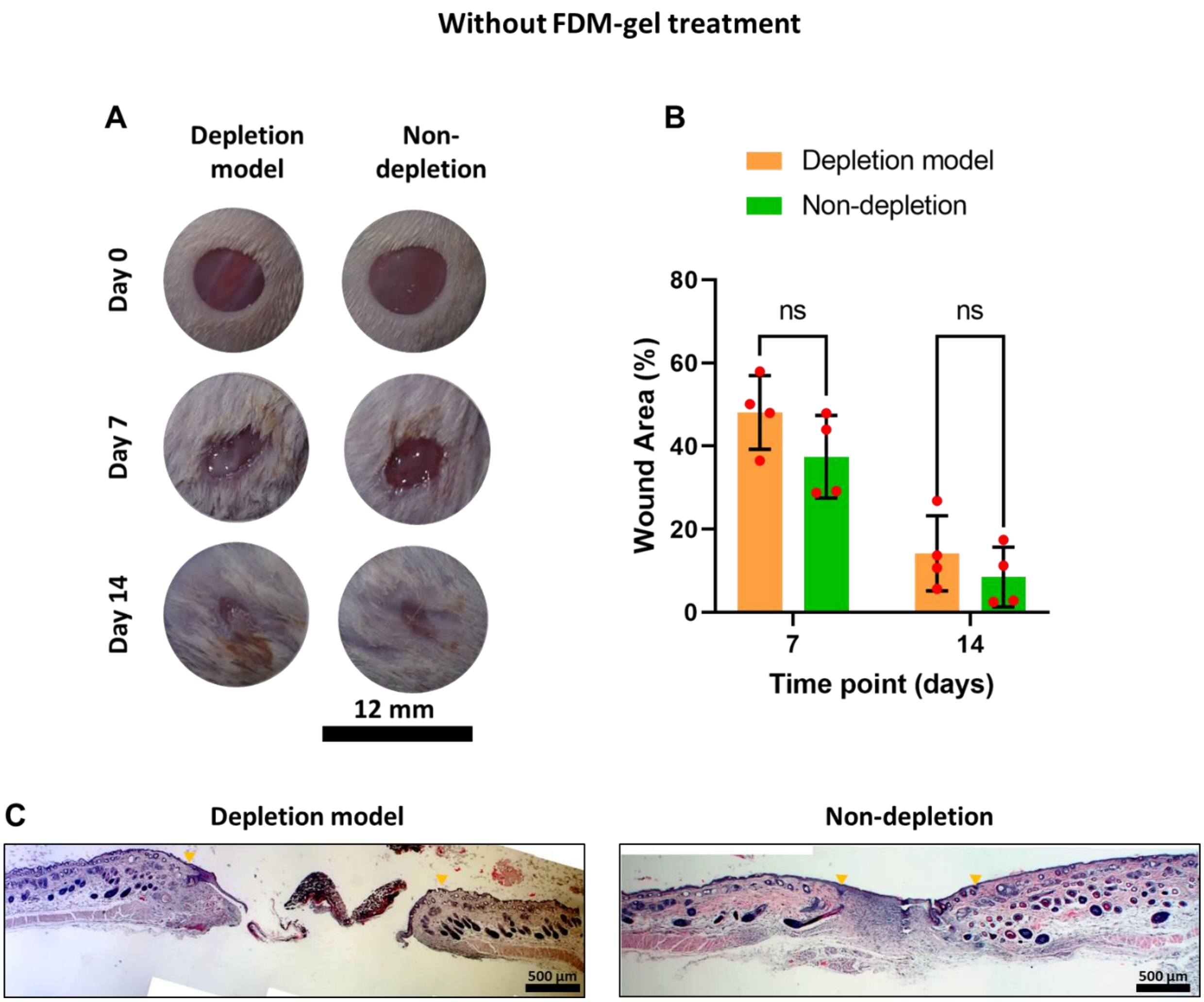

Fig. S12. Development of macrophage depletion mouse model by administering clodronate in the full-thickness skin wounds. No FDM-gel treatment at this time. (A) Gross appearance of the wounds treated with clodronate liposome (depletion model) or control liposome (non-depletion) at 7 and 14 days, respectively. (B) Quantitative measurement of the wound area (%) between macrophage depletion and non-depletion model group. (C) Histological observation of the wound regions (epidermis and dermis) via H&E staining.
